# Supplementary material for: Cofactors facilitate bona fide prion misfolding in vitro but are not necessary for the infectivity of recombinant murine prions
Source: PLoS Pathog. 2025 Jan 22;21(1):e1012890. doi: 10.1371/journal.ppat.1012890 (PMC11774496; doi:10.1371/journal.ppat.1012890)
Supplement: S1 Table — The mutated codon is highlighted in bold in all primers used for site-directed mutagenesis. Primers used for the 5’ and 3’ extremes in all cases are shown under the name L108 (wild-type), with the nucleotides belonging to the PrP ORF underlined. (PDF) [file ppat.1012890.s001.pdf]

S1 Table. List of forward (Fw) and reverse (Rv) primers used for the generation of the 20 mouse rec-PrP variants with all possible amino acids in position 108. The mutated codon is highlighted in bold in all primers used for site-directed mutagenesis. Primers used for the 5' and 3' extremes in all cases are shown under the name L108 (wild-type), with the nucleotides belonging to the PrP ORF underlined.

Name	Primer
L108A	Fw: 5'- CCAAAAACCAAC**GCG**AAGCATGTGGCAG -3'<br>Rv: 5'- CTGCCACATGCTT**CGC**GTTGGTTTTTGG -3'
L108C	Fw: 5'- CCAAAAACCAAC**TGC**AAGCATGTGGCAG -3'<br>Rv:5'- CTGCCACATGCTT**GCA**GTTGGTTTTTGG -3
L108D	Fw: 5'- CCAAAAACCAAC**GAC**AAGCATGTGGCAG -3'<br>Rv: 5'- CTGCCACATGCTT**GTC**GTTGGTTTTTGG -3'
L108E	Fw: 5'- 5'- CCAAAAACCAAC**GAA**AAGCATGTGGCAG -3'<br>Rv: 5'- CTGCCACATGCTT**TTC**GTTGGTTTTTGG -3'
L108F	Fw: 5'- GCAAACCAAAAACCAAC**TTC**AAGCATGTGGCAGGGG -3'<br>Rv: 5'- CCCCTGCCACATGCTT**GAA**GTTGGTTTTTGGTTTGC -3'
L108G	Fw: 5'- CCAAAAACCAAC**GGC**AAGCATGTGGCAG -3'<br>Rv: 5'- CTGCCACATGCTT**GCC**GTTGGTTTTTGG -3'
L108H	Fw: 5'- CCAAAAACCAAC**CAT**AAGCATGTGGCAG -3'<br>Rv: 5'- CTGCCACATGCTT**ATG**GTTGGTTTTTGG -3'
L108I	Fw: 5'- CCAAAAACCAAC**ATT**AAGCATGTGGCAGGGGC -3'<br>Rv: 5'- CTGCCACATGCTT**AAT**GTTGGTTTTTGGTTTGC -3'
L108K	Fw: 5'- CCAAAAACCAAC**AAA**AAGCATGTGGCAG -3'<br>Rv: 5'- CTGCCACATGCTT**TTT**GTTGGTTTTTGG -3'
L108	Fw: 5'- AGGAGATATACCATGAAAAAGCGGCCAAAGCCTGAA -3'<br>Rv: 5'- GTGATGGTGATGTTAGGATCTTCTCCCGTCGTAATA -3'
L108M	Fw: 5'- CCAAAAACCAAC**ATG**AAGCATGTGGCAG -3'<br>Rv: 5'- CTGCCACATGCTT**CAT**GTTGGTTTTTGG -3'
L108N	Fw: 5'- CCAAAAACCAAC**AAC**AAGCATGTGGCAG -3'<br>Rv: 5'- CTGCCACATGCTT**GTT**GTTGGTTTTTGG -3'
L108P	Fw: 5'- CCAAAAACCAAC**CCG**AAGCATGTGGCAG -3'<br>Rv: 5'- CTGCCACATGCTT**CGG**GTTGGTTTTTGG -3'
L108Q	Fw: 5'- CCAAAAACCAAC**CAG**AAGCATGTGGCAG -3'<br>Rv: 5'- CTGCCACATGCTT**CTG**GTTGGTTTTTGG -3'
L108R	Fw: 5'- CCAAAAACCAAC**CGC**AAGCATGTGGCAG -3'<br>Rv: 5'- CTGCCACATGCTT**GCG**GTTGGTTTTTGG -3'
L108S	Fw: 5'- CCAAAAACCAAC**AGC**AAGCATGTGGCAG -3'<br>Rv: 5'- CTGCCACATGCTT**GCT**GTTGGTTTTTGG -3'
L108T	Fw: 5'- CCAAAAACCAAC**ACC**AAGCATGTGGCAG -3'<br>Rv: 5'- CTGCCACATGCTT**GGT**GTTGGTTTTTGG -3'
L108V	Fw: 5'- CCAAAAACCAAC**GTG**AAGCATGTGGCAG -3'<br>Rv: 5'- CTGCCACATGCTT**CAC**GTTGGTTTTTGG -3'
L108W	Fw: 5'- CCAAAAACCAAC**TGG**AAGCATGTGGCAG -3'<br>Rv: 5'- CTGCCACATGCTT**CCA**GTTGGTTTTTGG -3'
L108Y	Fw: 5'- CCAAAAACCAAC**TAC**AAGCATGTGGCAG -3'<br>Rv: 5'- CTGCCACATGCTT**GTA**GTTGGTTTTTGG -3'
