# Supplementary material for: Cofactors facilitate bona fide prion misfolding in vitro but are not necessary for the infectivity of recombinant murine prions
Source: PLoS Pathog. 2025 Jan 22;21(1):e1012890. doi: 10.1371/journal.ppat.1012890 (PMC11774496; doi:10.1371/journal.ppat.1012890)
Supplement: S1 Fig — Substrates were evaluated to confirm comparable rec-PrP concentrations prior to assessing their spontaneous misfolding capacity. Except for the L108C variant, which formed dimers (indicated by an asterisk) due to potential disulfide bridge formation, all other variants exhibited similar concentrations and were deemed suitable for PMSA. Despite efforts to concentrate the L108C substrate, dimer formation limited its preparation. The assay proceeded with the available substrate, acknowledging that while the lower concentration might underestimate misfolding propensity, enhanced misfolding would remain detectable as misfolding is not significantly affected within a certain concentration range [1]. MW: Molecular weight marker. (PDF) [file ppat.1012890.s002.pdf]

Substrates for PMSA

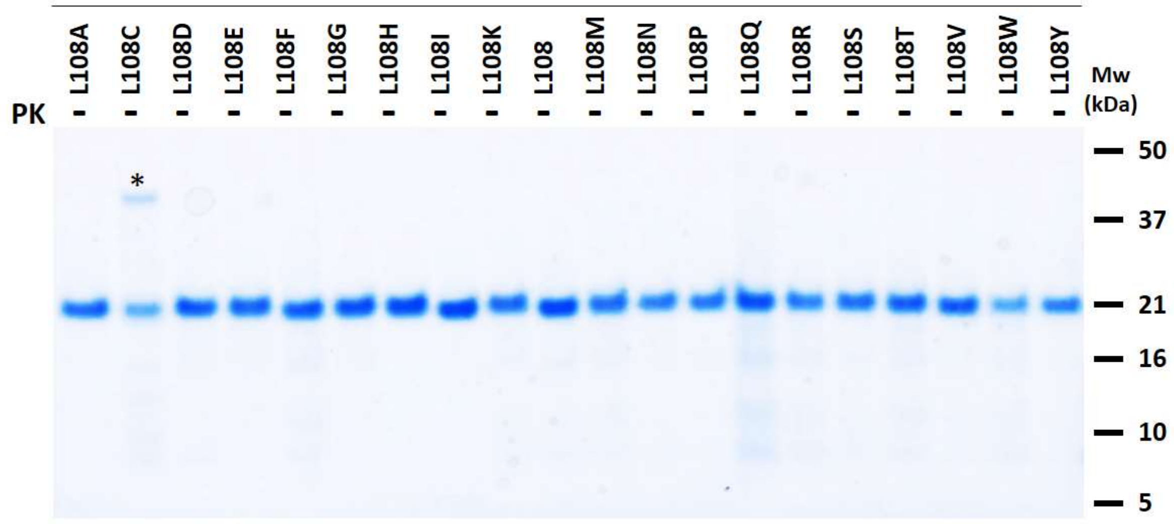

S1 Fig. Electrophoresis and total protein staining of the 20 PMSA substrates prepared with mouse PrP variants with all naturally occurring amino acids at position 108. Substrates were analyzed to verify comparable rec-PrP concentrations prior to assessing their spontaneous misfolding capacity. Except for the L108C variant, which formed dimers (indicated by an asterisk) due to potential disulfide bridge formation, all other variants exhibited similar concentrations and were considered suitable for PMSA. Despite efforts to concentrate the L108C substrate, dimer formation limited its effective preparation. The assay was performed with the available substrate, acknowledging that while the lower concentration might underestimate misfolding propensity, significant misfolding would still be detectable, as misfolding rates remain relatively stable within a certain concentration range [1]. MW: Molecular weight marker.

Reference

1 Erana H, Sampedro-Torres-Quevedo C, Charco JM, Diaz-Dominguez CM, Peccati F, San-Juan-Ansoleaga M, Vidal E, Goncalves-Anjo N, Perez-Castro MA, Gonzalez-Miranda Eet al (2024) A Protein Misfolding Shaking Amplification-based method for the spontaneous generation of hundreds of bona fide prions. Nat Commun 15: 2112 Doi 10.1038/s41467-024-46360-2
