# Supplementary material for: Cofactors facilitate bona fide prion misfolding in vitro but are not necessary for the infectivity of recombinant murine prions
Source: PLoS Pathog. 2025 Jan 22;21(1):e1012890. doi: 10.1371/journal.ppat.1012890 (PMC11774496; doi:10.1371/journal.ppat.1012890)
Supplement: S2 Fig — The relative resistance to PK digestion of the four PMSA products was evaluated to determine their prion-like characteristics and assess potential conformational differences. Products were digested with increasing PK concentrations (25–2000 µg/ml) at 42 °C for 45 minutes, followed by electrophoresis and protein staining. Additionally, three independent digestion reactions were performed and rec-PrPres levels evaluated through Western blot and densitometric analysis, the results of which are plotted and normalized using the samples digested with 25 µg/ml of PK as reference of maximum, 100%, signal intensity. Normalized data from the densitometric analysis, revealed similar resistance for stMI-01, btMI-05, and btMI-09, with no significant differences. In contrast, stMI-03 exhibited significantly lower resistance compared to stMI-01 (p = 0.013), btMI-05 (p = 0.013), and btMI-09 (p = 0.028), indicating distinct conformational properties. An undigested substrate control (rec-MoL108I) was included for size reference. MW: Molecular weight marker. B) Evaluation of the self-propagation capacity of the misfolded recombinant PMSA products in vitro in homologous and C) heterologous recombinant PrP-containing substrates. The ability of the PMSA products to induce misfolding of homologous mouse L108I recombinant PrP was tested via serial dilutions (10−1 to 10−11) in PMSA. All products propagated efficiently up to at least a 10−8 dilution, with btMI-09 reaching 10−11. PK-resistant misfolded rec-PrP was detected via digestion, electrophoresis, and total protein staining, with results displayed in shades of grey to indicate the percentage of positive replicates. For the evaluation of propagation capacity in a heterologous substrate, PMSA products were tested in mouse wild-type L108 recombinant PrP substrate (10−1 dilution, three replicates). Most seeds propagated in this heterologous substrate, confirming their prion-like properties. However, stMI-03 failed to propagate under these [file ppat.1012890.s003.pdf]

A

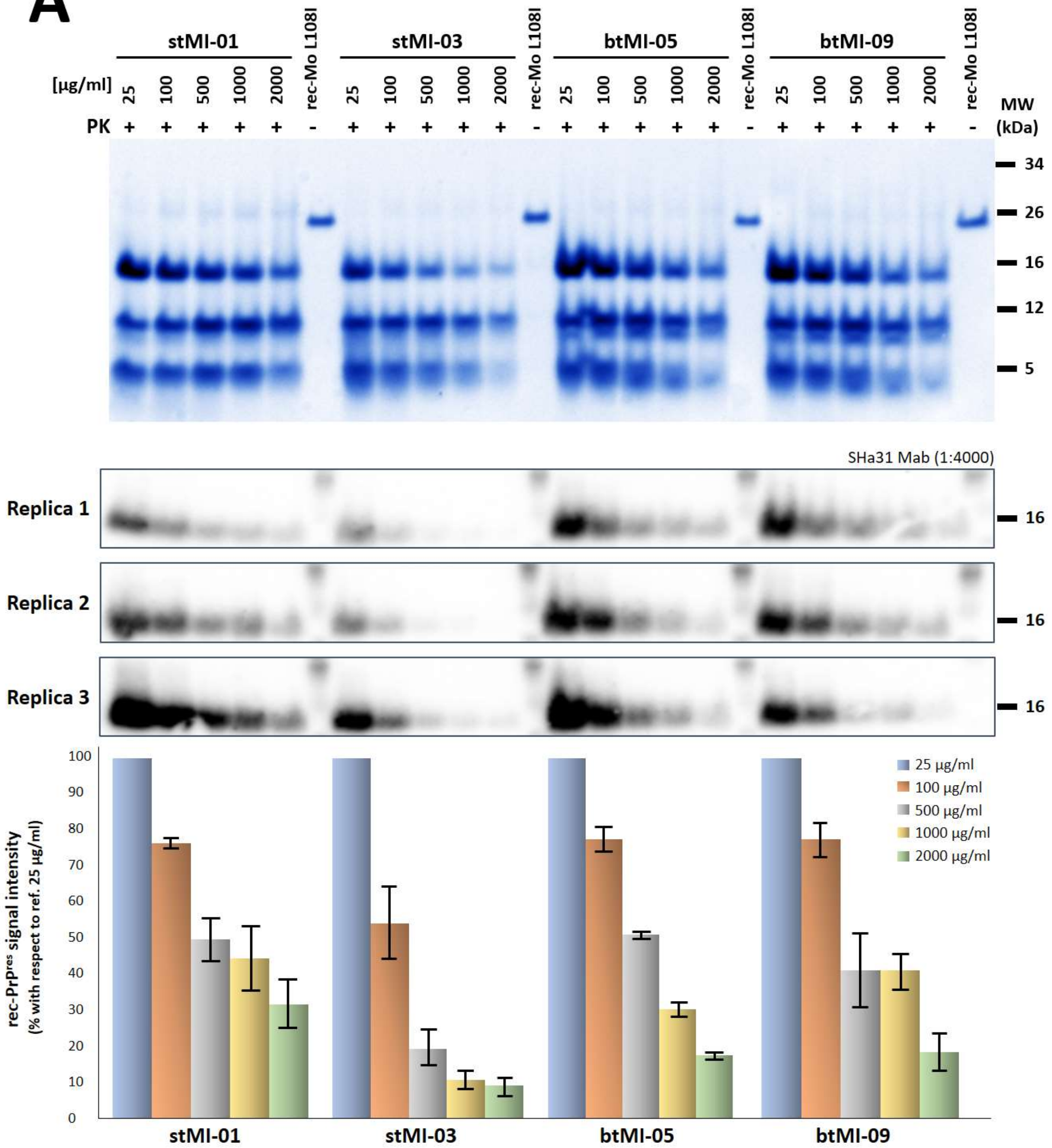

stMI-01
stMI-03
btMI-05
btMI-09
rec-Mo L108I
[µg/ml]
25
100
500
1000
2000
PK
+
-
MW (kDa)
34
26
16
12
5
SHa31 Mab (1:4000)
Replica 1
Replica 2
Replica 3
rec-PrPres signal intensity
(% with respect to ref. 25 µg/ml)
0
10
20
30
40
50
60
70
80
90
100
25 µg/ml
100 µg/ml
500 µg/ml
1000 µg/ml
2000 µg/ml

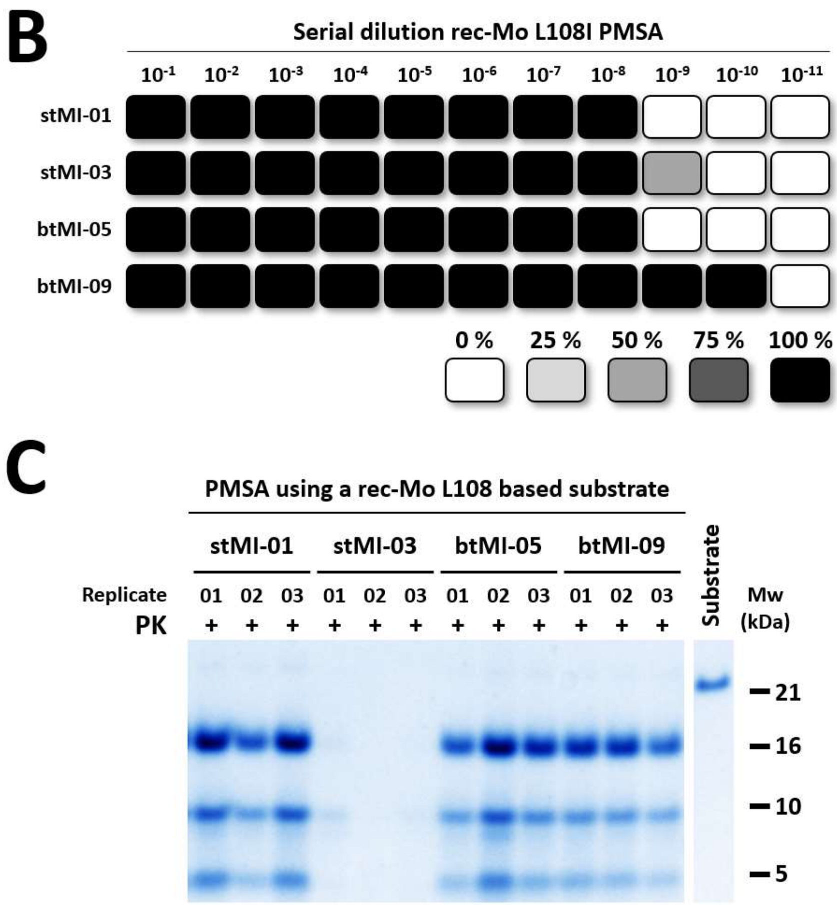

S2 Fig. A) Proteinase K resistance assay of PMSA products (stMI-01, stMI-03, btMI-05, btMI-09). The relative resistance to PK digestion of the four PMSA products was evaluated to determine their prion-like characteristics and assess potential conformational differences. Products were digested with increasing PK concentrations (25–2000 µg/ml) at 42 °C for 45 minutes, followed by electrophoresis and protein staining. Additionally, three independent digestion reactions were performed and rec-PrP^{res} levels evaluated through Western blot and densitometric analysis, the results of which are plotted and normalized using the samples digested with 25 µg/ml of PK as reference of maximum, 100 %, signal intensity. Normalized data from the densitometric analysis, revealed similar resistance for stMI-01, btMI-05, and btMI-09, with no significant differences. In contrast, stMI-03 exhibited significantly lower resistance compared to stMI-01 ($p = 0.013$), btMI-05 ($p = 0.013$), and btMI-09 ($p = 0.028$), indicating distinct conformational properties. An undigested substrate control (rec-MoL108I) was included for size reference. MW: Molecular weight marker. **B) Evaluation of the self-propagation capacity of the misfolded recombinant PMSA products *in vitro* in homologous and C) heterologous recombinant PrP-containing substrates.** The ability of the PMSA products to induce misfolding of homologous mouse L108I recombinant PrP was tested via serial dilutions (10^{-1} to 10^{-11}) in PMSA. All products propagated efficiently up to at least a 10^{-8} dilution, with btMI-09 reaching 10^{-10}. PK-resistant misfolded rec-PrP was detected via digestion, electrophoresis, and total protein staining, with results displayed in shades of grey to indicate the percentage of positive replicates. For the evaluation of propagation capacity in a heterologous substrate, PMSA products were tested in mouse wild-type L108 recombinant PrP substrate (10^{-1} dilution, three replicates). Most seeds propagated in this heterologous substrate, confirming their prion-like properties. However, stMI-03 failed to propagate under these conditions, likely due to strain-specific polymorphic barriers consistent with its differential PK resistance. PK: Proteinase K; MW: Molecular weight marker.
