# Supplementary material for: Cofactors facilitate bona fide prion misfolding in vitro but are not necessary for the infectivity of recombinant murine prions
Source: PLoS Pathog. 2025 Jan 22;21(1):e1012890. doi: 10.1371/journal.ppat.1012890 (PMC11774496; doi:10.1371/journal.ppat.1012890)
Supplement: S3 Fig — The images display sixteen representative micrographs for each of the PMSA products A) stMI-01, B) stMI-03, C) btMI-05, and D) btMI-09 after partial purification through ultracentrifugation in a density gradient. Partially purified samples were stained with uracil acetate and imaged with a transmission electron microscope JEM-1230 (JEOL) at 100 kV, equipped with a CCD Orius SC1000 (GATAN) camera. All four PMSA products show fibrillar structures reminiscent of brain-derived prion rods, with virtually indistinguishable ultrastructure. In all four cases, clusters of yet unidentified electrodense material (Em) were observed near the rods. Whether these are a contaminant from the purification process or biologically significant for fiber formation is yet to be determined. Notably, in the btMI-09 preparation (panel D), apart from the major fiber type found in the other preparations, two additional fibril populations were observed. The micrographs revealed some thinner fibers (Tf, thin fibers) and clusters of especially short rods (Sr, short rods) that were not observed in the other PMSA products. E) The most notable structural findings or shared elements among the four PMSA preparations have been highlighted in a selection of micrographs. In most fibers, two parallel axial densities of approximately 12 nm were observed, resembling rail tracks. In all preparations, straight (S, in orange) and curved (C, in yellow) fibers were detected, which could represent distinct fiber populations. Lateral clustering of fibers organized in bundles was another common finding in all the products analyzed. The presence of torsions (indicated by yellow arrows) in some fibers suggests a helicoidal symmetry, although most seemed flat or presented long half-pitches that precluded their identification. Each image in the group of sixteen contains a link that opens a higher resolution version in a web browser when clicked. (PDF) [file ppat.1012890.s004.pdf]

A

stMI-01

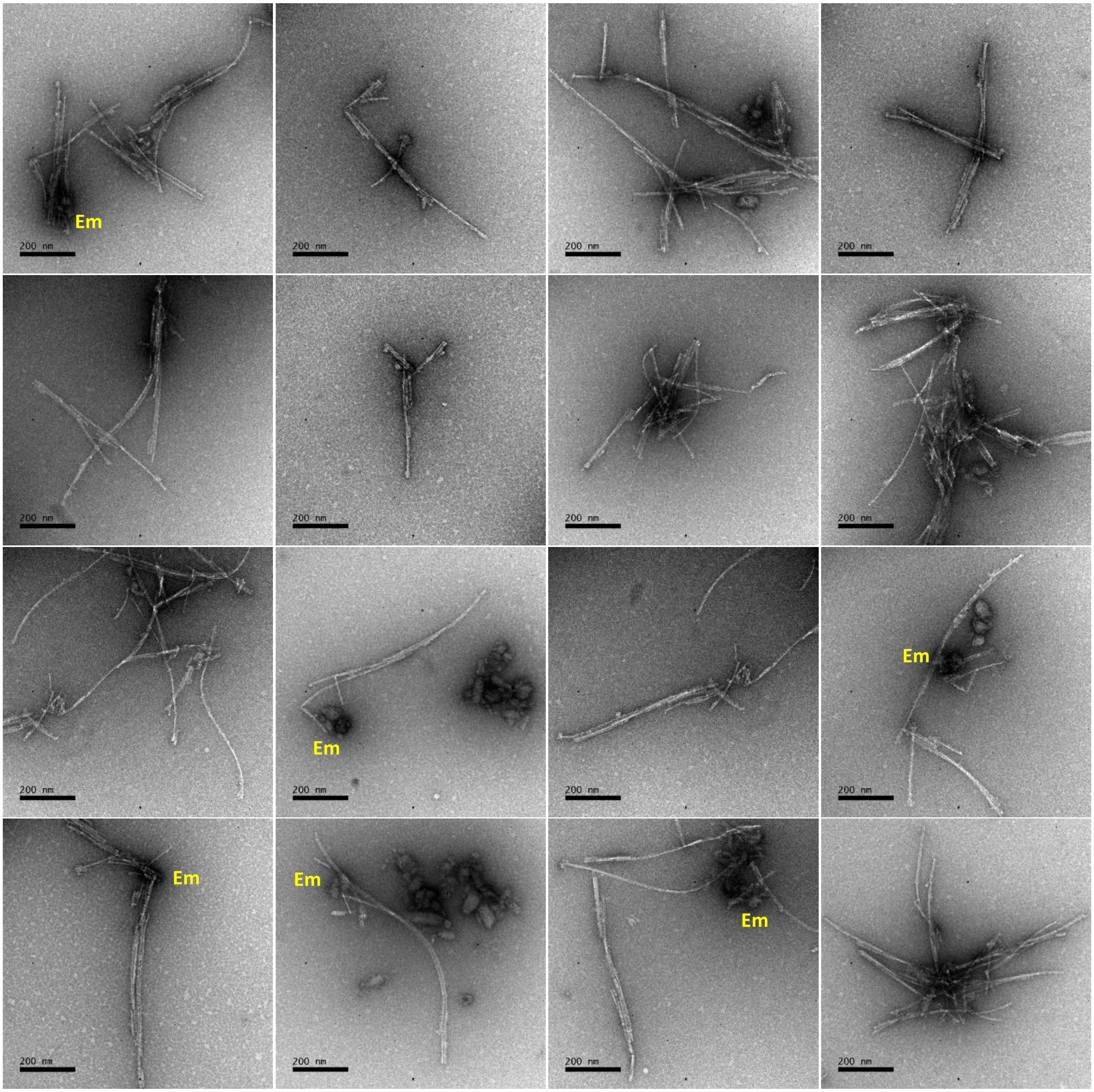

B

stMI-03

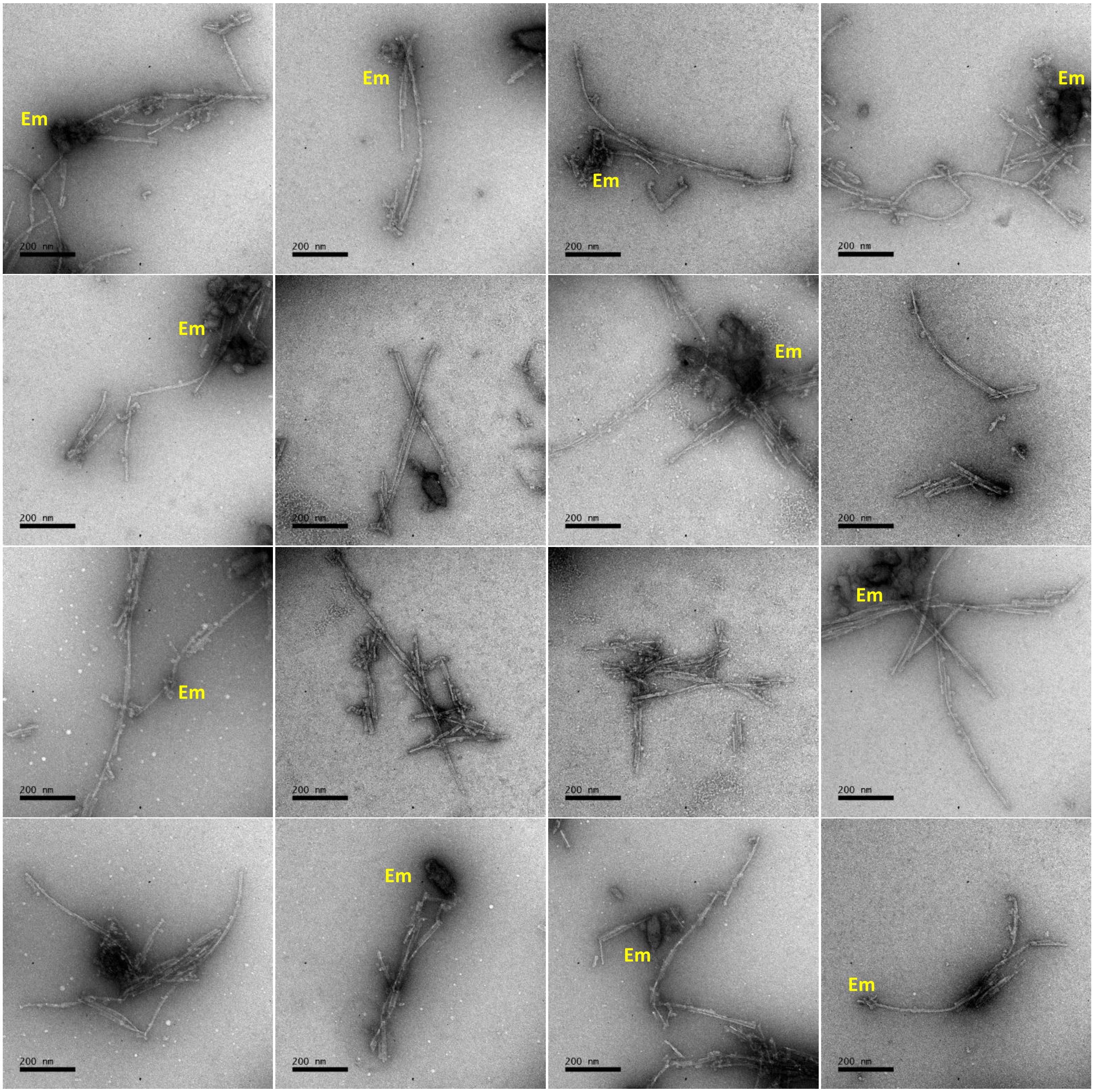
Em
200 nm
Em
200 nm
Em
200 nm
Em
200 nm
Em
200 nm
200 nm
Em
200 nm
200 nm
Em
200 nm
200 nm
200 nm
Em
200 nm
200 nm
Em
200 nm
Em
200 nm
Em
200 nm

C

btMI-05

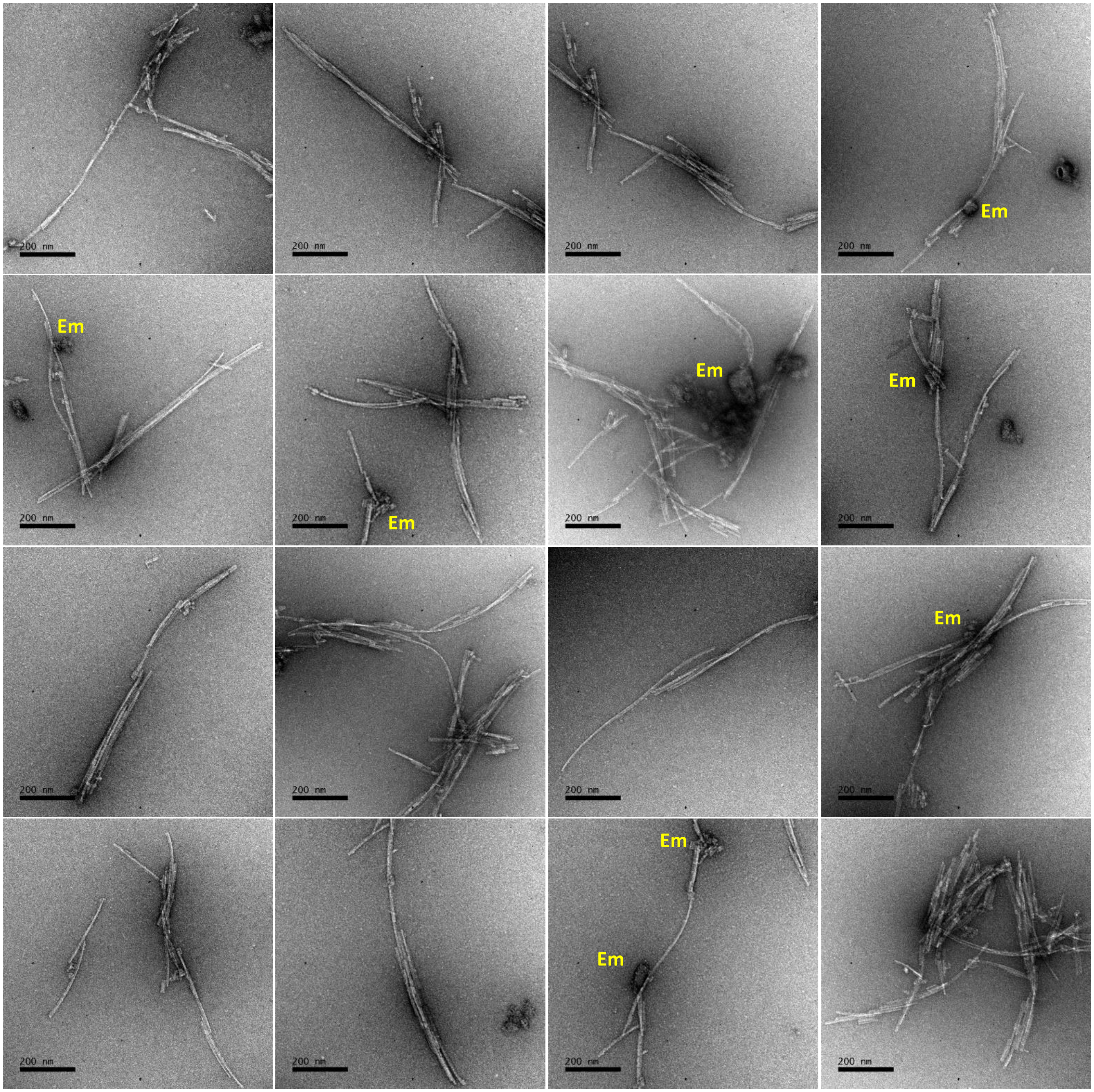

D

btMI-09

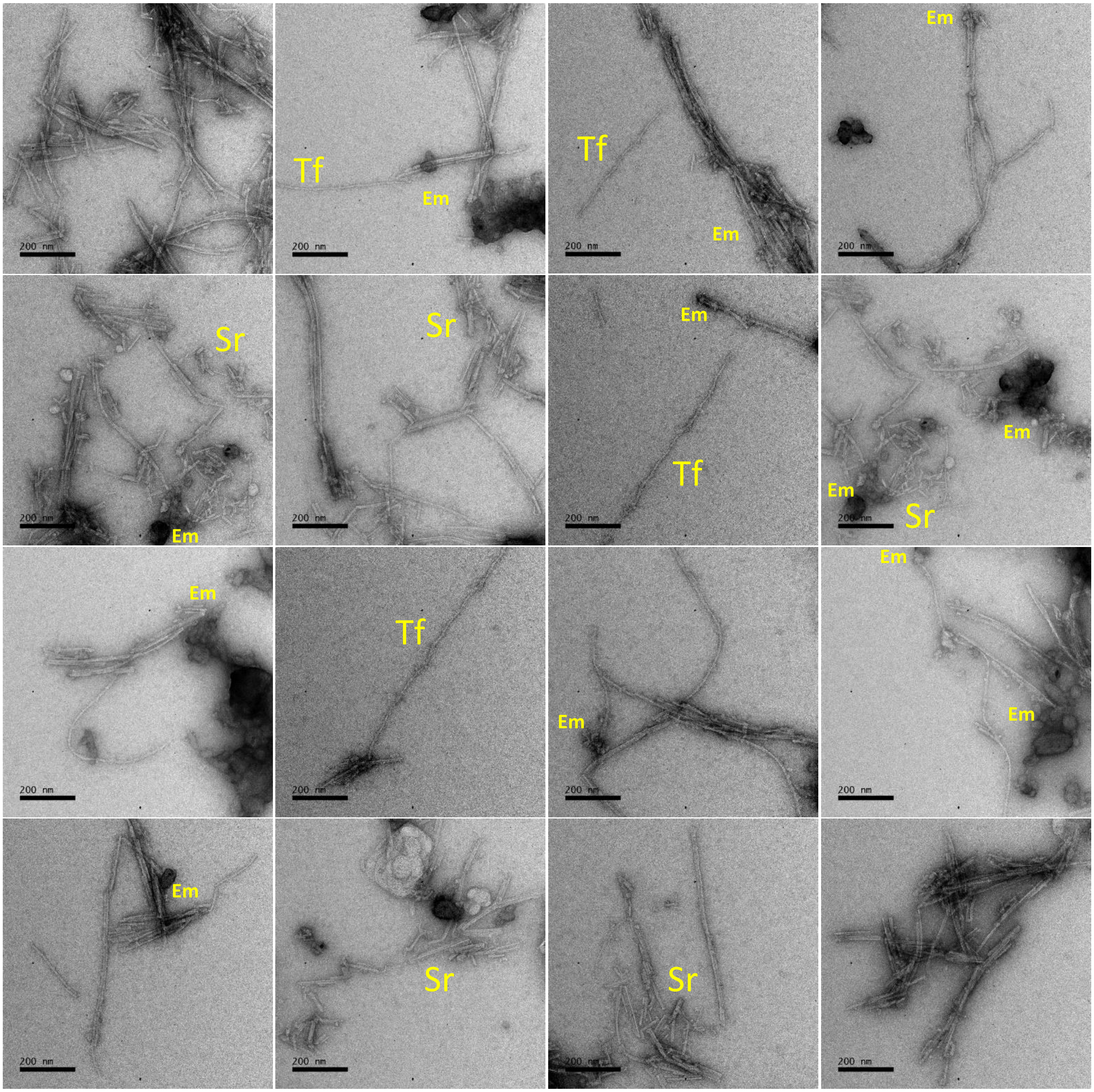
200 nm
Tf
Em
200 nm
Tf
Em
200 nm
Em
200 nm
Sr
200 nm
Em
Sr
200 nm
Em
Tf
200 nm
Em
Em
Sr
200 nm
Em
200 nm
Tf
200 nm
Em
200 nm
Em
Em
200 nm
Em
200 nm
Sr
200 nm
Sr
200 nm
200 nm

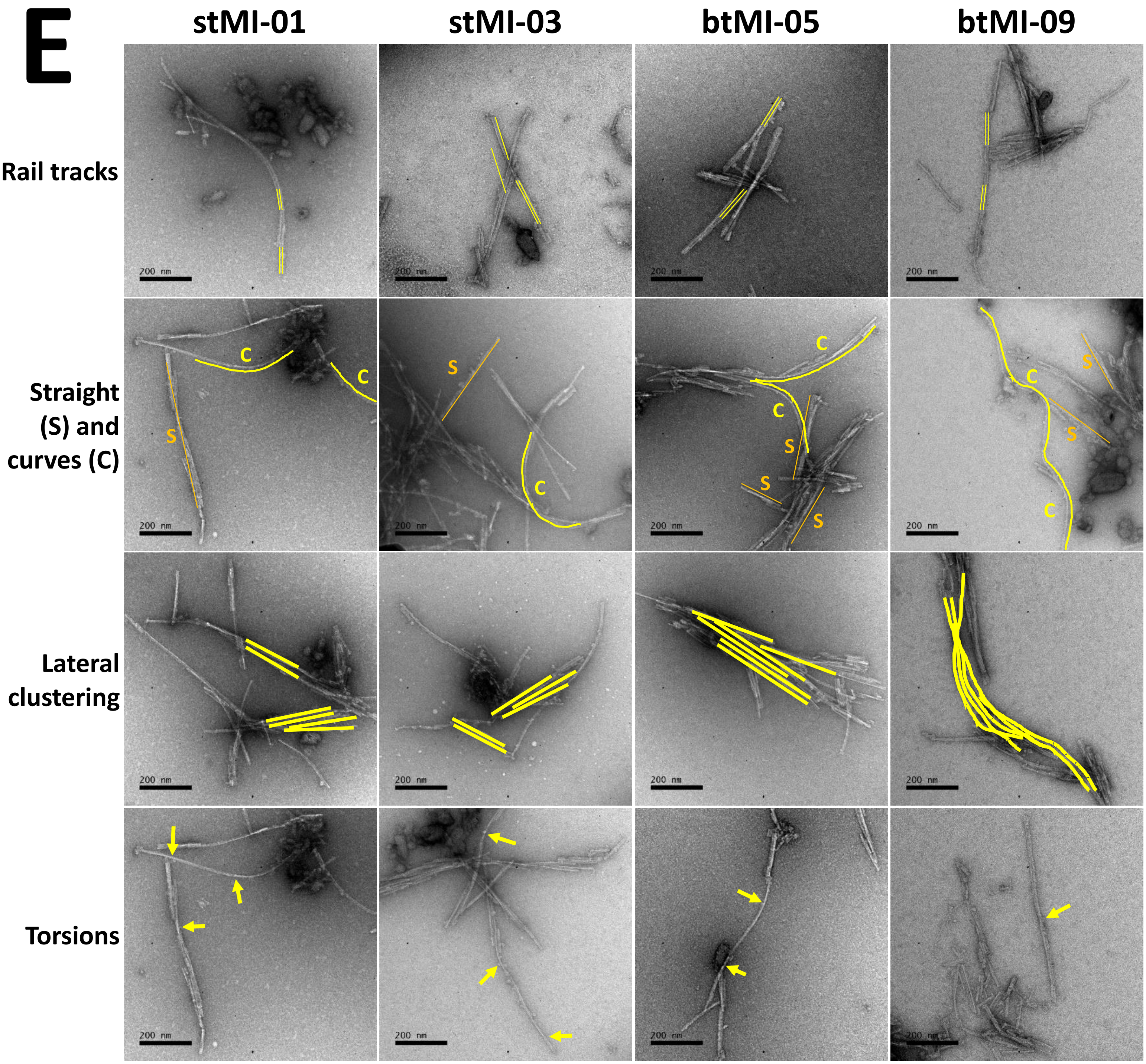
E
stMI-01
stMI-03
btMI-05
btMI-09
Rail tracks
Straight (S) and curves (C)
Lateral clustering
Torsions
200 nm
S
C

S3 Fig. Negative staining electron microscopy micrographs of the four PMSA products selected for further characterization. The images display sixteen representative micrographs for each of the PMSA products **A)** stMI-01, **B)** stMI-03, **C)** btMI-05, and **D)** btMI-09 after partial purification through ultracentrifugation in a density gradient. Partially purified samples were stained with uracil acetate and imaged with a transmission electron microscope JEM-1230 (JEOL) at 100 kV, equipped with a CCD Orius SC1000 (GATAN) camera. All four PMSA products show fibrillar structures reminiscent of brain-derived prion rods, with virtually indistinguishable ultrastructure. In all four cases, clusters of yet unidentified electrodense material (Em) were observed near the rods. Whether these are a contaminant from the purification process or biologically significant for fiber formation is yet to be determined. Notably, in the btMI-09 preparation (panel D), apart from the major fiber type found in the other preparations, two additional fibril populations were observed. The micrographs revealed some thinner fibers (Tf, thin fibers) and clusters of especially short rods (Sr, short rods) that were not observed in the other PMSA products. **E)** The most notable structural findings or shared elements among the four PMSA preparations have been highlighted in a selection of micrographs. In most fibers, two parallel axial densities of approximately 12 nm were observed, resembling rail tracks. In all preparations, straight (S, in orange) and curved (C, in yellow) fibers were detected, which could represent distinct fiber populations. Lateral clustering of fibers organized in bundles was another common finding in all the products analyzed. The presence of torsions (indicated by yellow arrows) in some fibers suggests a helicoidal symmetry, although most seemed flat or presented long half-pitches that precluded their identification. Each image in the group of sixteen contains a link that opens a higher resolution version in a web browser when clicked.
