# Supplementary material for: Cofactors facilitate bona fide prion misfolding in vitro but are not necessary for the infectivity of recombinant murine prions
Source: PLoS Pathog. 2025 Jan 22;21(1):e1012890. doi: 10.1371/journal.ppat.1012890 (PMC11774496; doi:10.1371/journal.ppat.1012890)
Supplement: S4 Fig — To predict the potential infectivity in vivo of the distinct PMSA products selected for further characterization (stMI-01 Dx, stMI-03 Dx, btMI-05 Dx and btMI-09 Dx), their capacity to induce misfolding of PrPC in brain homogenates of TgMoL108I animals, expressing 3-fold the mouse L108I PrP, was evaluated using PMCA. PMSA products were partially purified by ultracentrifugation through a density gradient, resulting in visible halos of proteic aggregates in all four samples. These purified fractions exhibited indistinguishable biochemical properties after proteinase K digestion and retained the same electrophoretic pattern as the original product. These purified fractions were used to seed a PMCA substrate based on TgMoL108I brain homogenate at 1:10 dilutions, and a 24 h PMCA reaction was performed (R1). Two additional serial PMCA rounds were conducted, with the second round (R2) seeded at 1:10 dilution using the product from the first round, and the third round (R3) utilizing a 1:10 dilution of the product form the second one. After the three serial PMCA rounds of 24 h, PrPSc detection was carried out by proteinase K digestion and Western blotting (mAb Sha31 at a dilution of 1:4,000). A seeded tube at 1:10 but not submitted to PMCA is also included for each PMSA product, referred to as R0, to show the signal corresponding to the recombinant seed, slightly lower than the unglycosylated PrPC from brain. Finally, an unseeded tube was also included together with each PMSA product in every PMCA round, performing also serial passages as control for cross-contamination or spontaneous misfolding. All four recombinant seeds were able to misfold brain-derived PrPC from the first PMCA round, giving rise to the classical three-banded PrPSc pattern, suggesting the potential infectivity of these preparations in vivo. PK: Proteinase K; NBH control: Normal brain homogenate from TgMoL108I. Mw: Molecular weight. (PDF) [file ppat.1012890.s005.pdf]

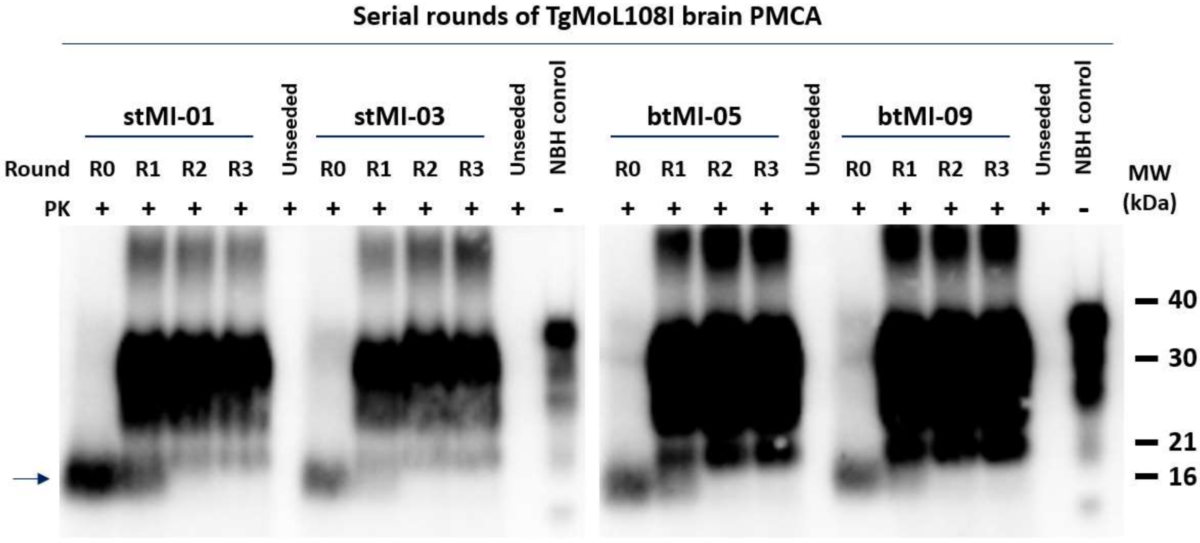

S4 Fig. Assessment of the capacity of the four selected recombinant misfolded PrP generated by PMSA to induce misfolding of PrP^{C} from brain *in vitro*. To predict the potential infectivity *in vivo* of the distinct PMSA products selected for further characterization (stMI-01 Dx, stMI-03 Dx, btMI-05 Dx and btMI-09 Dx), their capacity to induce misfolding of PrP^{C} in brain homogenates of TgMoL108I animals, expressing 3-fold the mouse L108I PrP, was evaluated using PMCA. PMSA products were partially purified by ultracentrifugation through a density gradient, resulting in visible halos of proteic aggregates in all four samples. These purified fractions exhibited indistinguishable biochemical properties after proteinase K digestion and retained the same electrophoretic pattern as the original product. These purified fractions were used to seed a PMCA substrate based on TgMoL108I brain homogenate at 1:10 dilutions, and a 24 h PMCA reaction was performed (R1). Two additional serial PMCA rounds were conducted, with the second round (R2) seeded at 1:10 dilution using the product from the first round, and the third round (R3) utilizing a 1:10 dilution of the product form the second one. After the three serial PMCA rounds of 24 h, PrP^{Sc} detection was carried out by proteinase K digestion and Western blotting (mAb Sha31 at a dilution of 1:4,000). A seeded tube at 1:10 but not submitted to PMCA is also included for each PMSA product, referred to as R0, to show the signal corresponding to the recombinant seed, slightly lower than the unglycosylated PrP^{C} from brain. Finally, an unseeded tube was also included together with each PMSA product in every PMCA round, performing also serial passages as control for cross-contamination or spontaneous misfolding. All four recombinant seeds were able to misfold brain-derived PrP^{C} from the first PMCA round, giving rise to the classical three-banded PrP^{Sc} pattern, suggesting the potential infectivity of these preparations *in vivo*. PK: Proteinase K; NBH control: Normal brain homogenate from TgMoL108I. Mw: Molecular weight.
