# Supplementary material for: Cofactors facilitate bona fide prion misfolding in vitro but are not necessary for the infectivity of recombinant murine prions
Source: PLoS Pathog. 2025 Jan 22;21(1):e1012890. doi: 10.1371/journal.ppat.1012890 (PMC11774496; doi:10.1371/journal.ppat.1012890)
Supplement: S5 Fig — Histopathological assessment of spongiform lesions and PrPres deposits of C57BL/6 mice after serial inoculation (1st and 2nd passages) with brain homogenate from a terminal ill TgMoL108I mouse with spontaneous disease. Analysis revealed no spongiform changes upon hematoxylin and eosin staining (H&E), as shown in the thalamic region of representative animals. PrPres deposits were undetectable, using 6C2 mAb (1:1,000), demonstrating that this spontaneously generated prion strain failed to transmit to wild type mice. H&E: Hematoxylin and eosin; IHC: Immunohistochemistry. (PDF) [file ppat.1012890.s006.pdf]

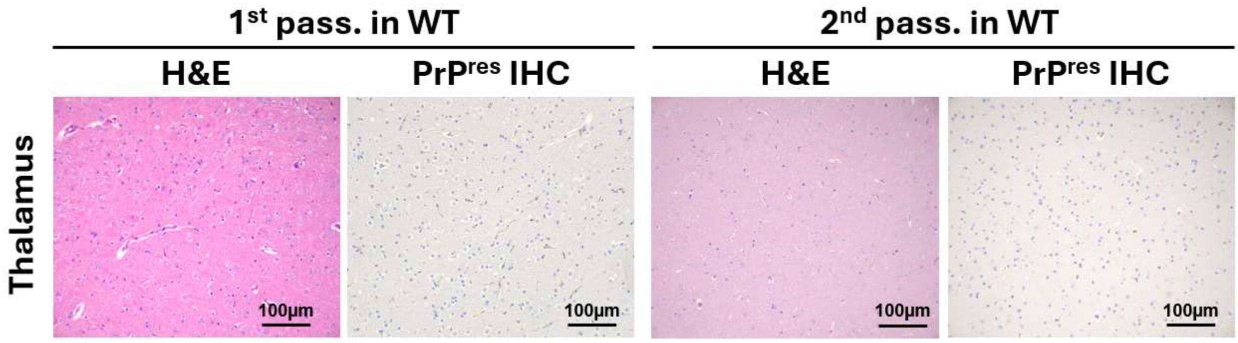

S5 Fig. Anatomopathological analysis of C57BL/6 mice inoculated intracerebrally with brain homogenate from a spontaneously sick TgMoL108I transgenic mouse. Histopathological assessment of spongiform lesions and PrP^{res} deposits of C57BL/6 mice after serial inoculation (1st and 2nd passages) with brain homogenate from a terminal ill TgMoL108I mouse with spontaneous disease. Analysis revealed no spongiform changes upon hematoxylin and eosin staining (H&E), as shown in the thalamic region of representative animals. PrP^{res} deposits were undetectable, using 6C2 mAb (1:1,000), demonstrating that this spontaneously generated prion strain failed to transmit to wild type mice. H&E: Hematoxylin and eosin; IHC: Immunohistochemistry.
