# Supplementary material for: Cofactors facilitate bona fide prion misfolding in vitro but are not necessary for the infectivity of recombinant murine prions
Source: PLoS Pathog. 2025 Jan 22;21(1):e1012890. doi: 10.1371/journal.ppat.1012890 (PMC11774496; doi:10.1371/journal.ppat.1012890)
Supplement: S6 Fig — Histopathological assessment of spongiform lesions and PrPres deposits of PMSA preparations stMI-03, btMI-05 and btMI-09 after secondary transmission into C57BL/6 mice(which was unsuccessful already at first passage for stMI-01) shows moderate to intense spongiform changes upon hematoxylin and eosin staining (H&E), as can be seen in the thalamic region of representative animals from each group. PrPres deposits, labeled with 6C2 mAb (1:1,000), were detectable but very faint, all showing punctate or granular deposits associated with glia or neurons and in the neuropil (see digitally enlarged images on the left). Spongiform lesion profiles and PrPres deposition profiles, shown on the right, represent the mean semi-quantitative scoring (0–4, vertical axis, ± standard error of the mean -error bars-) of the spongiform lesions (continuous line, black) and the immunohistochemical labelling of PrPres deposits (dashed line, black) against 14 brain regions. Although there are some differences, mostly in terms of spongiform lesion intensity, stMI-03, btMI-05, and btMI-09-inoculated animals show highly coincident lesions and PrPres staining, suggesting infection by the same strain or the convergence of the recombinant preparations in vivo to the same strain through selection or adaptation. H&E: Hematoxylin and eosin staining; IHC: Immunohistochemistry. (DOCX) [file ppat.1012890.s007.docx]

**
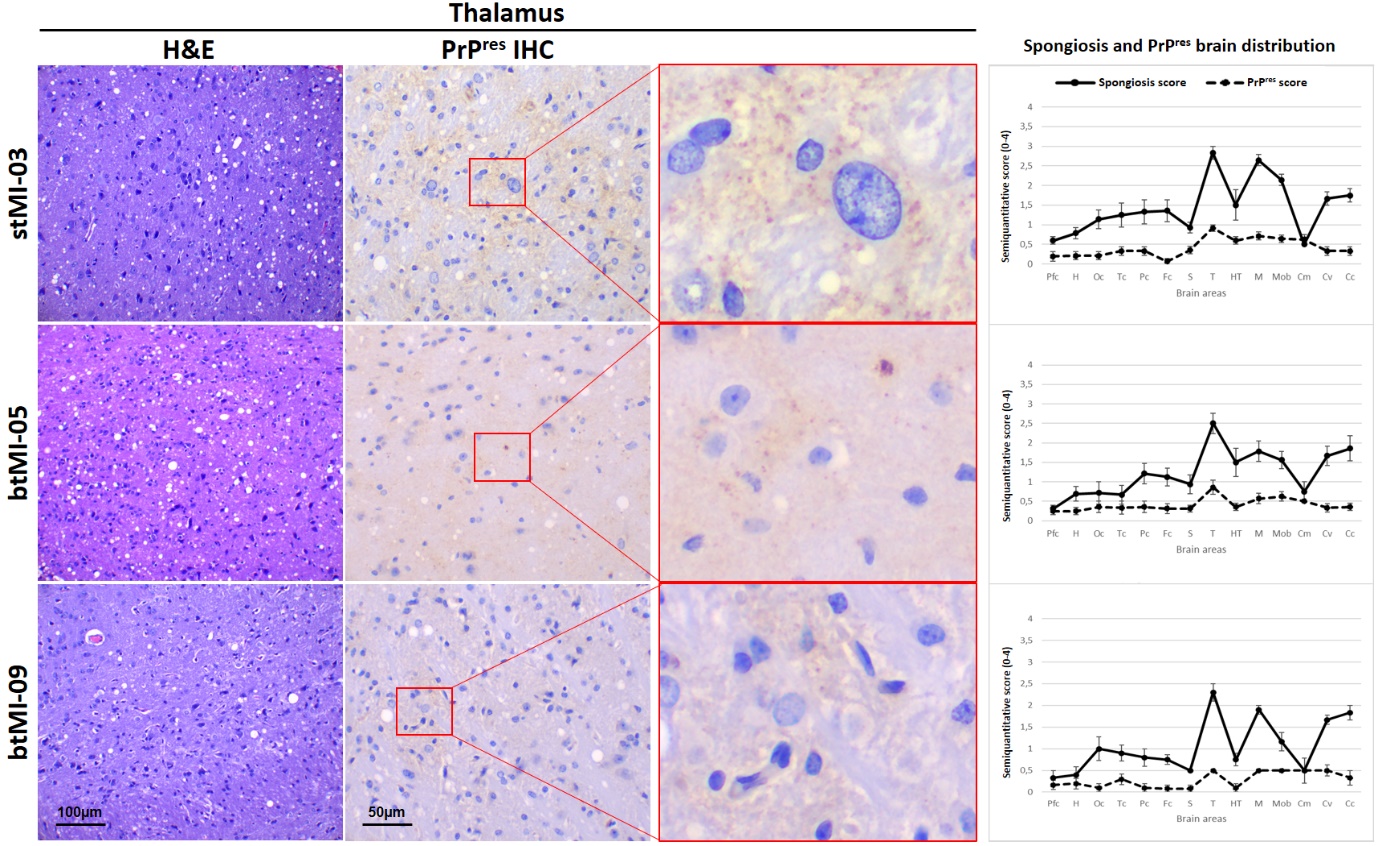
**

**S6 Fig. Brain lesion and PrP^res^ deposit distribution of the wild-type mice-passaged recombinant PMSA products after secondary transmission in wild-type mice.** Histopathological assessment of spongiform lesions and PrP^res^ deposits of PMSA preparations stMI-03, btMI-05 and btMI-09 after secondary transmission into C57BL/6 mice, (which was unsuccessful already at first passage for stMI-01) shows moderate to intense spongiform changes upon hematoxylin and eosin staining (H&E), as can be seen in the thalamic region of representative animals from each group. PrP^res^ deposits, labeled with 6C2 mAb (1:1,000), were detectable but very faint, all showing punctate or granular deposits associated with glia or neurons and in the neuropil (see digitally enlarged images on the left). Spongiform lesion profiles and PrP^res^ deposition profiles, shown on the right, represent the mean semi-quantitative scoring (0–4, vertical axis, ± standard error of the mean -error bars-) of the spongiform lesions (continuous line, black) and the immunohistochemical labelling of PrP^res^ deposits (dashed line, black) against 14 brain regions (Pfc: piriform cortex, H: hippocampus, Oc: occipital cortex, Tc: temporal cortex, Pc: parietal cortex, Fc: frontal cortex, cc: corpus callosum; S: striatum, T: thalamus, HT: hypothalamus, M: mesencephalon, Mob: medulla oblongata, Cm: cerebellar nuclei, Cv: cerebellar vermis, Cc: cerebellar cortex). Although there are some differences, mostly in terms of spongiform lesion intensity, stMI-03, btMI-05, and btMI-09-inoculated animals show highly coincident lesions and PrP^res^ staining, suggesting infection by the same strain or the convergence of the recombinant preparations *in vivo* to the same strain through selection or adaptation. H&E: Hematoxylin and eosin staining; IHC: Immunohistochemistry.
