# Supplementary material for: Cofactors facilitate bona fide prion misfolding in vitro but are not necessary for the infectivity of recombinant murine prions
Source: PLoS Pathog. 2025 Jan 22;21(1):e1012890. doi: 10.1371/journal.ppat.1012890 (PMC11774496; doi:10.1371/journal.ppat.1012890)
Supplement: S7 Fig — A) Biochemical analysis of TgVole (1x) brains inoculated with four selected PMSA products. Brain homogenates from all inoculated animals showing clinical signs of transmissible spongiform encephalopathy were analyzed by proteinase K (PK) digestion, electrophoresis, and Western blot (Sha31, 1:4,000). Results revealed the presence of classical three-banded pattern PrPSc, demonstrating the infectious capacity and bona fide nature of the recombinant prions generated spontaneously by PMSA and their cross-species transmissibility. The gel shows two representative samples from each group inoculated with the distinct recombinant products and one of the brain-derived RML and 22L prions, also passaged in TgVole (1x). All the samples are indistinguishable from each other except for those from stMI-03 inoculated animals, among which two electrophoretic patterns could be found, one of them showing a lower unglycolsylated band. B) Kaplan-Meier survival curves of TgVole (1x) inoculated with PMSA products and classical murine prion strains. Kaplan-Meier survival curves illustrate the incubation periods following intracerebral inoculations with the distinct PMSA products, RML, and 22L. stMI-01 shows great dispersion in incubation periods, while btMI-05 and btMI-09 exhibit profiles more similar to brain-derived strains RML and 22L, with lower dispersion but in the case of btMI-09 with a shorter incubation period, indicating its’ high cross-species transmissibility. Groups showing greater dispersion might indicate stronger barriers due to strain characteristics, mixtures of conformers undergoing slightly different selection process in each animal, or the formation of unstable or somewhat immature conformers, although likely due to the high susceptibility of bank vole PrPC to misfolding, most of the inocula show much lower dispersion than for previous models. PK: Proteinase K; NBH: Undigested normal brain homogenate; MW: Molecular weight marker. (PDF) [file ppat.1012890.s008.pdf]

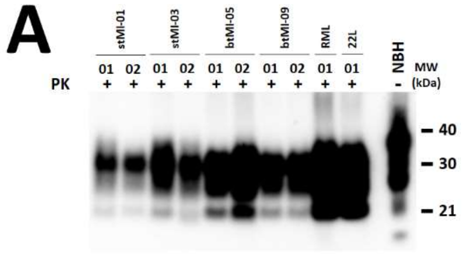

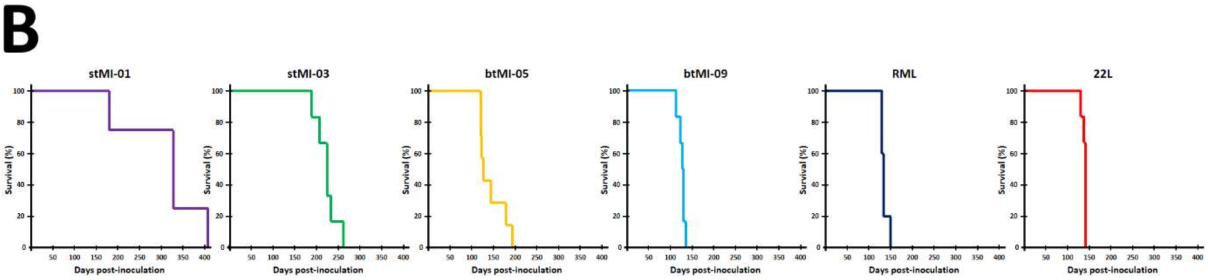

S7 Fig. Biochemical analysis and survival curves of TgVole (1x) mice inoculated with spontaneously misfolded PMSA products. A) Biochemical analysis of TgVole (1x) brains inoculated with four selected PMSA products. Brain homogenates from all inoculated animals showing clinical signs of transmissible spongiform encephalopathy were analyzed by proteinase K (PK) digestion, electrophoresis, and Western blot (Sha31, 1:4,000). Results revealed the presence of classical three-banded pattern PrP^{Sc}, demonstrating the infectious capacity and *bona fide* nature of the recombinant prions generated spontaneously by PMSA and their cross-species transmissibility. The gel shows two representative samples from each group inoculated with the distinct recombinant products and one of the brain-derived RML and 22L prions, also passaged in TgVole (1x). All the samples are indistinguishable from each other except for those from stMI-03 inoculated animals, among which two electrophoretic patterns could be found, one of them showing a lower unglycolsylated band. **B) Kaplan-Meier survival curves of TgVole (1x) inoculated with PMSA products and classical murine prion strains**. Kaplan-Meier survival curves illustrate the incubation periods following intracerebral inoculations with the distinct PMSA products, RML, and 22L. stMI-01 shows great dispersion in incubation periods, while btMI-05 and btMI-09 exhibit profiles more similar to brain-derived strains RML and 22L, with lower dispersion but in the case of btMI-09 with a shorter incubation period, indicating its' high cross-species transmissibility. Groups showing greater dispersion might indicate stronger barriers due to strain characteristics, mixtures of conformers undergoing slightly different selection process in each animal, or the formation of unstable or somewhat immature conformers, although likely due to the high susceptibility of bank vole PrP^{C} to misfolding, most of the inocula show much lower dispersion than for previous models. PK: Proteinase K; NBH: Undigested normal brain homogenate; MW: Molecular weight marker.
