# Supplementary figures and images for: Cofactors facilitate bona fide prion misfolding in vitro but are not necessary for the infectivity of recombinant murine prions
Source: PLoS Pathog. 2025 Jan 22;21(1):e1012890. doi: 10.1371/journal.ppat.1012890 (PMC11774496; doi:10.1371/journal.ppat.1012890)

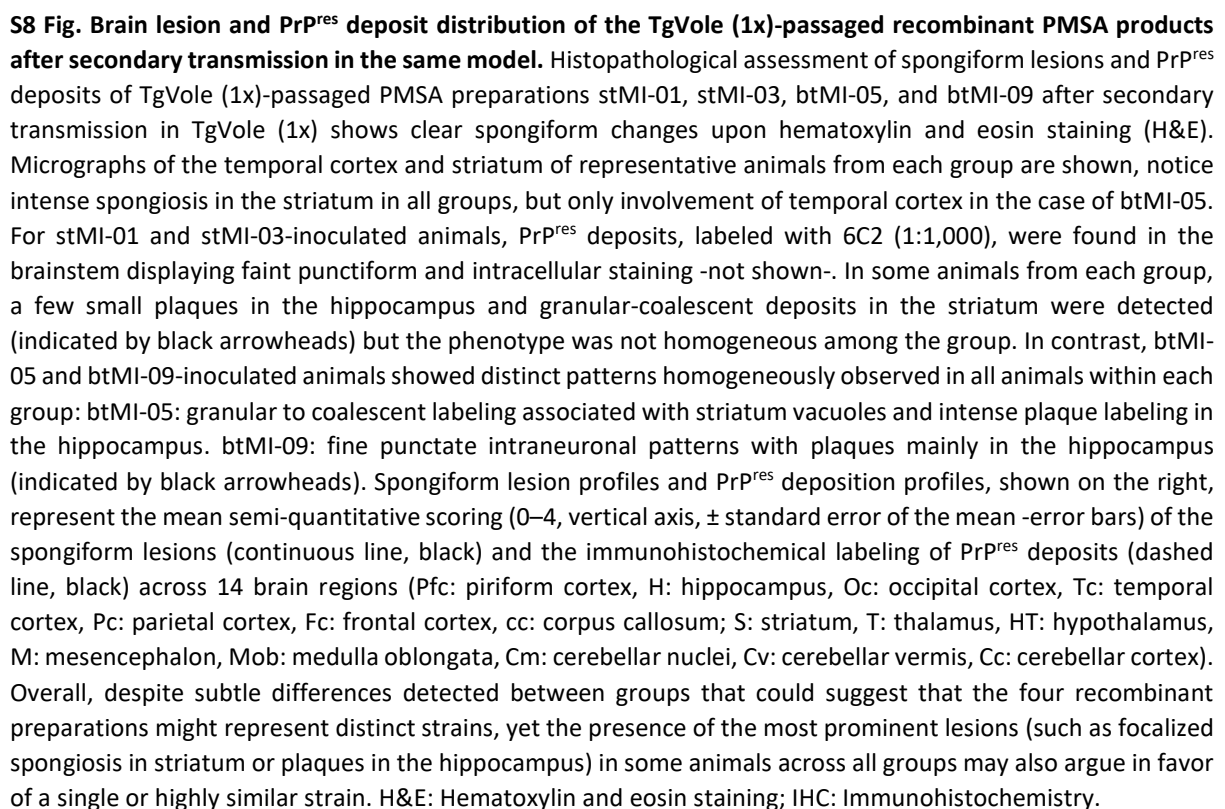

Supplement: S8 Fig — Histopathological assessment of spongiform lesions and PrPres deposits of TgVole (1x)-passaged PMSA preparations stMI-01, stMI-03, btMI-05, and btMI-09 after secondary transmission in TgVole (1x) shows clear spongiform changes upon hematoxylin and eosin staining (H&E). Micrographs of the temporal cortex and striatum of representative animals from each group are shown, notice intense spongiosis in the striatum in all groups, but only involvement of temporal cortex in the case of btMI-05. For stMI-01 and stMI-03-inoculated animals, in some animals from each group, a few small plaques in the hippocampus and granular-coalescent deposits in the striatum were detected (indicated by black arrowheads) but the phenotype was not homogeneous among the group. In contrast, btMI-05 and btMI-09-inoculated animals showed distinct patterns homogeneously observed in all animals within each group: btMI-05: granular to coalescent labeling associated with striatum vacuoles and intense plaque labeling in the hippocampus. btMI-09: fine punctate intraneuronal patterns with plaques mainly in the hippocampus (indicated by black arrowheads). Spongiform lesion profiles and PrPres deposition profiles, shown on the right, represent the mean semi-quantitative scoring (0–4, vertical axis, ± standard error of the mean -error bars) of the spongiform lesions (continuous line, black) and the immunohistochemical labeling of PrPres deposits (dashed line, black) across 14 brain regions (Pfc: piriform cortex, H: hippocampus, Oc: occipital cortex, Tc: temporal cortex, Pc: parietal cortex, Fc: frontal cortex, cc: corpus callosum; S: striatum, T: thalamus, HT: hypothalamus, M: mesencephalon, Mob: medulla oblongata, Cm: cerebellar nuclei, Cv: cerebellar vermis, Cc: cerebellar cortex). Overall, despite subtle differences detected between groups that could suggest that the four recombinant preparations might represent distinct strains, yet the presence of the most prominent lesions (such as focalized spongiosis [file ppat.1012890.s009.pdf]
