# Supplementary material for: Cofactors facilitate bona fide prion misfolding in vitro but are not necessary for the infectivity of recombinant murine prions
Source: PLoS Pathog. 2025 Jan 22;21(1):e1012890. doi: 10.1371/journal.ppat.1012890 (PMC11774496; doi:10.1371/journal.ppat.1012890)
Supplement: S9 Fig — Brain homogenates from all inoculated animals showing clinical signs of transmissible spongiform encephalopathy were analyzed by proteinase K (PK) digestion, electrophoresis, and Western blot (Sha31, 1:4,000). Results revealed the presence of classical three-banded pattern PrPSc, demonstrating the infectious capacity and bona fide nature of the recombinant prions generated spontaneously by PMSA and their cross-species transmissibility. The gel shows two representative samples from each group inoculated with the distinct recombinant products and one of the brain-derived RML as control of a classical prion strain. PrPSc from all three models inoculated with btMI-09 CB are indistinguishable from each other except for the amount of PrPSc, higher in wild-type likely due to their long incubation period. stMI-03 inoculated TgVole (1x) animals, are characterized by a lower molecular weight unglycosylated band, that could correspond to the selection of one of the two distinct conformers hinted previously for stMI-03 dex upon inoculation in TgMoL108I. PK: Proteinase K; NBH: Undigested normal brain homogenate; MW: Molecular weight marker. (PDF) [file ppat.1012890.s010.pdf]

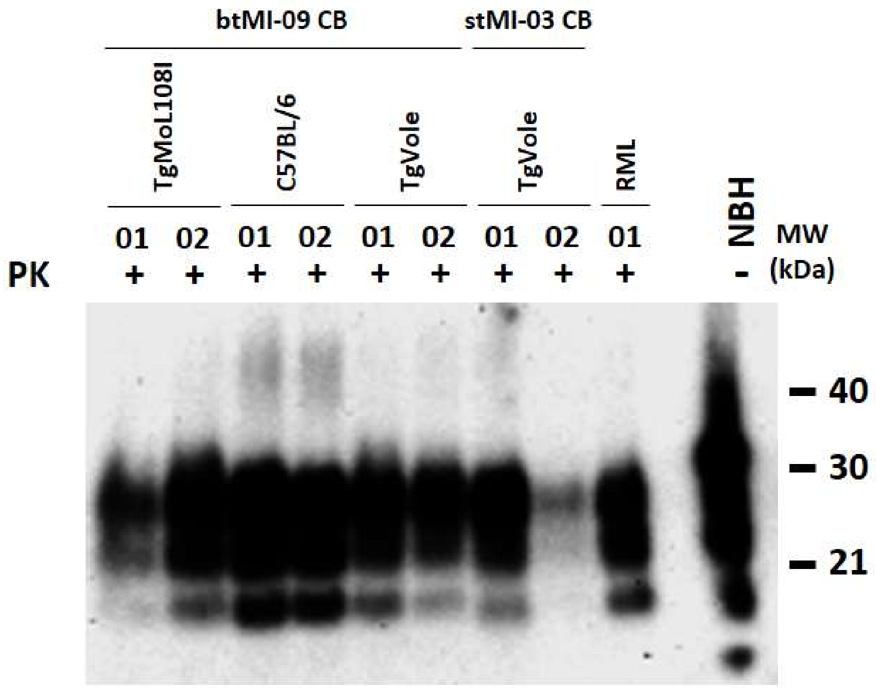

S9 Fig. Biochemical analysis of TgMoL108I, wild-type and TgVole (1x) mice brains inoculated with spontaneously misfolded PMSA products. Brain homogenates from all inoculated animals showing clinical signs of transmissible spongiform encephalopathy were analyzed by proteinase K (PK) digestion, electrophoresis, and Western blot (Sha31, 1:4,000). Results revealed the presence of classical three-banded pattern PrP^{Sc}, demonstrating the infectious capacity and *bona fide* nature of the recombinant prions generated spontaneously by PMSA and their cross-species transmissibility. The gel shows two representative samples from each group inoculated with the distinct recombinant products and one of the brain-derived RML as control of a classical prion strain. PrP^{Sc} from all three models inoculated with btMI-09 CB are indistinguishable from each other except for the amount of PrP^{Sc}, higher in wild-type likely due to their long incubation period. stMI-03 inoculated TgVole (1x) animals, are characterized by a lower molecular weight unglycosylated band, that could correspond to the selection of one of the two distinct conformers hinted previously for stMI-03 dex upon inoculation in TgMoL108I. PK: Proteinase K; NBH: Undigested normal brain homogenate; MW: Molecular weight marker.
