# Supplementary material for: Cofactors facilitate bona fide prion misfolding in vitro but are not necessary for the infectivity of recombinant murine prions
Source: PLoS Pathog. 2025 Jan 22;21(1):e1012890. doi: 10.1371/journal.ppat.1012890 (PMC11774496; doi:10.1371/journal.ppat.1012890)
Supplement: S10 Fig — A) Spongiform lesion (continuous line, black) and PrPres deposition (dashed line, grey) profiles were scored semi-quantitatively (0–4) across 14 brain regions, revealing distinct transmission patterns. stMI-03 CB successfully induced disease only in TgVole (1x) mice, showing mild and localized striatal spongiform lesions that differed from the more severe lesions caused by stMI-03 dex. Conversely, btMI-09 CB induced disease in all models with variations in lesion profiles. In TgMoL108I, btMI-09 CB caused milder hippocampal but more severe cerebellar lesions compared to btMI-09 dex, with consistent PrPres deposits patterns. In wild-type mice, btMI-09 CB induced strong spongiform lesions in brainstem and cortices with minimal PrPres labeling. In TgVole (1x), btMI-09 CB caused mild lesions with intraneuronal PrPres deposits in brainstem, cerebellar cortex, and hippocampus. B) A more detailed comparison of the lesions from wild-type mice inoculated with btMI-09 dex and btMI-09 CB illustrates the slightly higher severity of spongiform lesions in the latter through hematoxylin and eosin staining (H&E) of the thalamic region, and the similarity of the mild granular PrPres deposit pattern (see digitally enhanced sections on the left), labeled with 6C2 (1:1,000). Images of the btMI-09 dex-inoculated mice are the same as those used in S5 Fig to facilitate comparison. When plotted together, the spongiform lesion profile similarity between btMI-09 dex (red line) and btMI-09 CB (black line) is evident and suggests conservation of the pathobiological features of the original preparation in the cofactor-devoid version. C) Conversely, in TgVole (1x) mice inoculated with btMI-09 CB and btMI-09 dex shows the main differences observed in this model, namely the severe and focalized spongiform lesions, evident upon H&E staining, and PrPres plaques, labeled with 6C2 (1:1,000), in the striatum of the dextran-complemented preparation, which are absent in btMI-09 CB-inoculated animals. Imag [file ppat.1012890.s011.pdf]

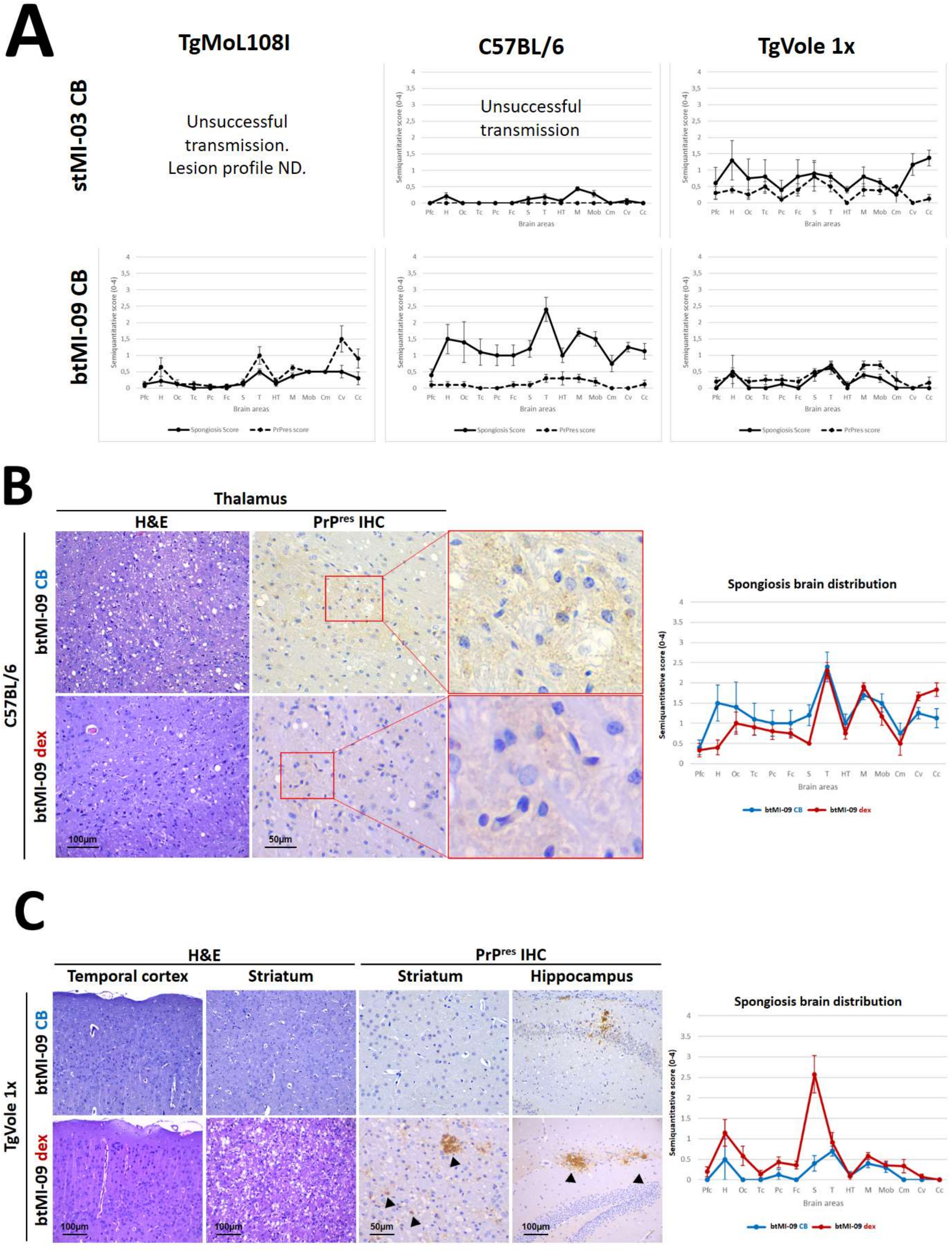

S10 Fig. Brain lesion and PrP^{res} deposition induced by recombinant prions stMI-03 and btMI-09 propagated in cofactor-devoid environment (CB) were compared to their original dextran sulfate-supplemented preparations across three animal models (TgMoL108I, wild-type, and TgVole (1x) mice). A) Spongiform lesion (continuous line, black) and PrP^{res} deposition (dashed line, grey) profiles were scored semi-quantitative (0–4) across 14 brain regions, revealing distinct transmission patterns. stMI-03 CB successfully induced disease only in TgVole (1x) mice, showing mild and localized striatal spongiform lesions that differed from the more severe

lesions caused by stMI-03 dex. Conversely, btMI-09 CB induced disease in all models with variations in lesion profiles. In TgMoL108I, btMI-09 CB caused milder hippocampal but more severe cerebellar lesions compared to btMI-09 dex, with consistent PrP^{res} deposits patterns. In wild-type mice, btMI-09 CB induced strong spongiform lesions in brainstem and cortices with minimal PrP^{res} labeling. In TgVole (1x), btMI-09 CB caused mild lesions with intraneuronal PrP^{res} deposits in brainstem, cerebellar cortex, and hippocampus. **B)** A more detailed comparison of the lesions from wild-type mice inoculated with btMI-09 dex and btMI-09 CB illustrates the slightly higher severity of spongiform lesions in the latter through hematoxylin and eosin staining (H&E) of the thalamic region, and the similarity of the mild granular PrP^{res} deposit pattern (see digitally enhanced sections on the left), labeled with 6C2 (1:1,000). Images of the btMI-09 dex-inoculated mice are the same as those used in S5 Fig to facilitate comparison. When plotted together, the spongiform lesion profile similarity between btMI-09 dex (red line) and btMI-09 CB (black line) is evident and suggests conservation of the pathobiological features of the original preparation in the cofactor-devoid version. **C)** Conversely, in TgVole (1x) mice inoculated with btMI-09 CB and btMI-09 dex shows the main differences observed in this model, namely the severe and focalized spongiform lesions, evident upon H&E staining, and PrP^{res} plaques, labeled with 6C2 (1:1,000), in the striatum of the dextran-complemented preparation, which are absent in btMI-09 CB-inoculated animals. Images of the btMI-09 dex-inoculated TgVole (1x) mice are the same as those used in S7 Fig to facilitate comparison. In this case, when plotted together, the spongiform lesion profile (± standard error of the mean -error bars-) clearly displays the differences found for the two preparations (btMI-09 dex in red and btMI-09 CB in black) in this model. H&E: Hematoxylin and eosin staining; IHC: Immunohistochemistry.
