# Supplementary material for: Cofactors facilitate bona fide prion misfolding in vitro but are not necessary for the infectivity of recombinant murine prions
Source: PLoS Pathog. 2025 Jan 22;21(1):e1012890. doi: 10.1371/journal.ppat.1012890 (PMC11774496; doi:10.1371/journal.ppat.1012890)
Supplement: S11 Fig — Histopathological assessment of spongiform lesions and PrPres deposits of TgMoL108I mice inoculated with the PMSA preparations btMI-09 dex, btMI-09 CB, btMI-09 dex2, and btMI-09 CB2. These preparations are products of the sequential adaptation of the original btMI-09 dex recombinant prion in cofactor-devoid and dextran sulfate-complemented PMSA substrates. The assessment aimed to compare the pathobiological features of all four preparations. A) Hematoxylin and eosin staining (H&E) shows moderate spongiform lesions in the thalamus and absence of spongiosis in the cerebellar cortex. The pattern of PrPres deposits, labeled with 2G11 monoclonal antibody (1:100), reveals PrPres small aggregates in the thalamus (indicated by black arrowheads) and larger plaques, characteristically located in the white matter of the cerebellar cortex. Spongiosis distribution profiles and PrPres deposition profiles for each group, shown below, represent the mean semi-quantitative scoring (0–4, vertical axis, ± standard error of the mean -error bars-) of the spongiform lesions (continuous line, black) and the immunohistochemical labeling of PrPres deposits (dashed line, black) across 14 brain regions. B) Spongiform lesion and PrPres deposit profiles plotted together (btMI-09 dex in yellow, btMI-09 CB in grey, btMI-09 dex2 in black, and btMI-09 CB2 in black) illustrate the high similarity of localization and intensity of the spongiform lesions and PrPres deposits in all cases. This suggests no significant alteration of the pathobiological features of the original btMI-09 preparation in this model occur related to the presence or absence of the cofactor in the propagation environment. H&E: Hematoxylin and eosin staining; IHC: Immunohistochemistry. (PDF) [file ppat.1012890.s012.pdf]

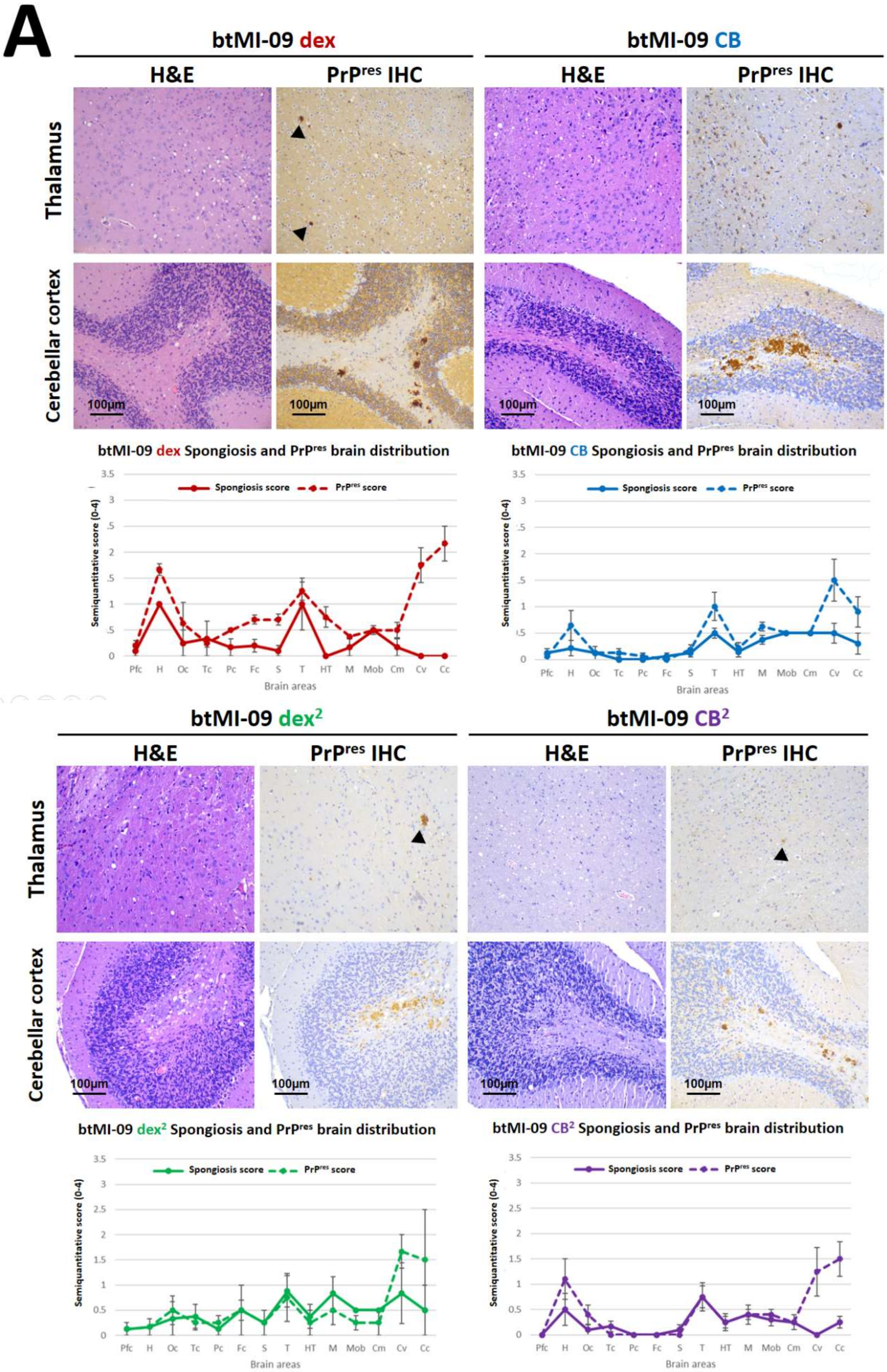
A
btMI-09 dex
btMI-09 CB
H&E
PrPres IHC
Thalamus
Cerebellar cortex
100µm
btMI-09 dex Spongiosis and PrPres brain distribution
btMI-09 CB Spongiosis and PrPres brain distribution
Spongiosis score
PrPres score
Semiquantitative score (0-4)
Pfc H Oc Tc Pc Fc S T HT M Mob Cm Cv Cc
Brain areas
btMI-09 dex2
btMI-09 CB2
btMI-09 dex2 Spongiosis and PrPres brain distribution
btMI-09 CB2 Spongiosis and PrPres brain distribution

# B

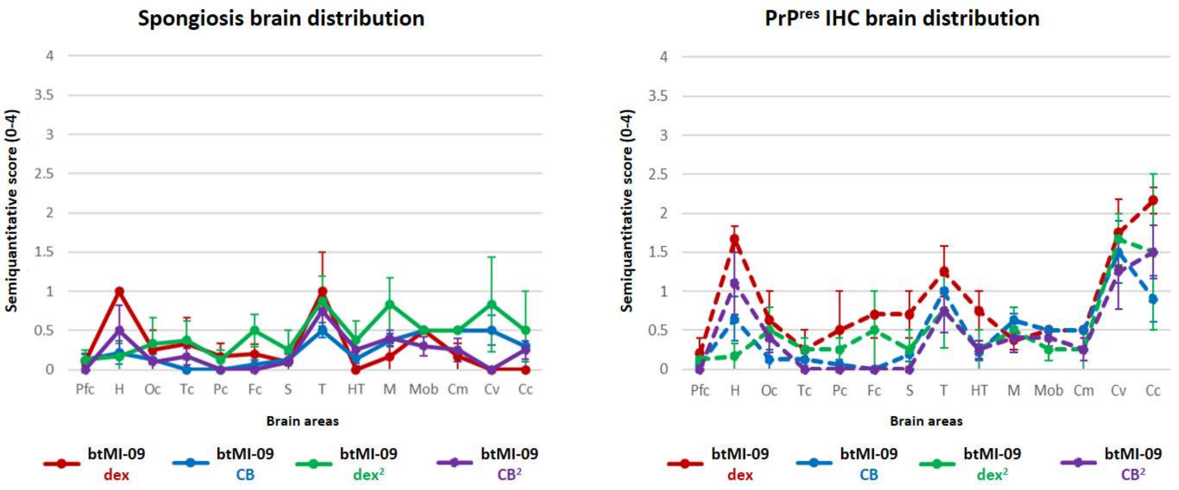


**S11 Fig. Brain lesion and PrP$^{res}$ deposit distribution in TgMoL108I mice inoculated with btMI-09 prion sequentially adapted across cofactor-devoid and dextran sulfate-complemented PMSA substrates.** Histopathological assessment of spongiform lesions and PrP$^{res}$ deposits of TgMoL108I mice inoculated with the PMSA preparations btMI-09 dex, btMI-09 CB, btMI-09 dex$^2$, and btMI-09 CB$^2$. These preparations are products of the sequential adaptation of the original btMI-09 dex recombinant prion in cofactor-devoid and dextran sulfate-complemented PMSA substrates. The assessment aimed to compare the pathobiological features of all four preparations. **A)** Hematoxylin and eosin staining (H&E) shows moderate spongiform lesions in the thalamus and absence of spongiosis in the cerebellar cortex. The pattern of PrP$^{res}$ deposits, labeled with 2G11 monoclonal antibody (1:100), reveals PrP$^{res}$ small aggregates in the thalamus (indicated by black arrowheads) and larger plaques, characteristically located in the white matter of the cerebellar cortex. Spongiosis distribution profiles and PrP$^{res}$ deposition profiles for each group, shown below, represent the mean semi-quantitative scoring (0–4, vertical axis, ± standard error of the mean -error bars-) of the spongiform lesions (continuous line, black) and the immunohistochemical labeling of PrP$^{res}$ deposits (dashed line, black) across 14 brain regions (Pfc: piriform cortex, H: hippocampus, Oc: occipital cortex, Tc: temporal cortex, Pc: parietal cortex, Fc: frontal cortex, cc: corpus callosum; S: striatum, T: thalamus, HT: hypothalamus, M: mesencephalon, Mob: medulla oblongata, Cm: cerebellar nuclei, Cv: cerebellar vermis, Cc: cerebellar cortex). **B)** Spongiform lesion and PrP$^{res}$ deposit profiles plotted together (btMI-09 dex in yellow, btMI-09 CB in grey, btMI-09 dex$^2$ in black, and btMI-09 CB$^2$ in black) illustrate the high similarity of localization and intensity of the spongiform lesions and PrP$^{res}$ deposits in all cases. This suggests no significant alteration of the pathobiological features of the original btMI-09 preparation in this model occur related to the presence or absence of the cofactor in the propagation environment. H&E: Hematoxylin and eosin staining; IHC: Immunohistochemistry.
