# Supplementary material for: Cofactors facilitate bona fide prion misfolding in vitro but are not necessary for the infectivity of recombinant murine prions
Source: PLoS Pathog. 2025 Jan 22;21(1):e1012890. doi: 10.1371/journal.ppat.1012890 (PMC11774496; doi:10.1371/journal.ppat.1012890)
Supplement: S12 Fig — The images display four representative micrographs for each of the following PMSA products: stMI-03 dex (original preparation), stMI-03 CB, adapted to a cofactor-free substrate, btMI-09 dex (original preparation), btMI-09 CB (adapted to a cofactor-free substrate), btMI-09 dex2 (resulting from the back-passage of btMI-09 CB to a dextran-complemented substrate), and btMI-09 CB2 (the product of readapting btMI-09 dex2 to a substrate without cofactor). Samples were partially purified through ultracentrifugation in a density gradient, stained with uranyl acetate, and imaged with a transmission electron microscope JEM-1230 (JEOL) at 100 kV, equipped with a CCD Orius SC1000 (GATAN) camera. All PMSA products show fibrillar structures reminiscent of brain-derived prion rods, with no notable differences from the original dextran-complemented products that could explain the observed changes in biochemical and biological features. In all cases, the most remarkable structural features previously observed are conserved upon propagation in different substrates, including rods with two parallel axial densities, presence of straight and curved fibers, unidentified electrodense material, and high propensity for lateral clustering. Each image in the group of four contains a link that opens a higher resolution version in a web browser when clicked. (PDF) [file ppat.1012890.s013.pdf]

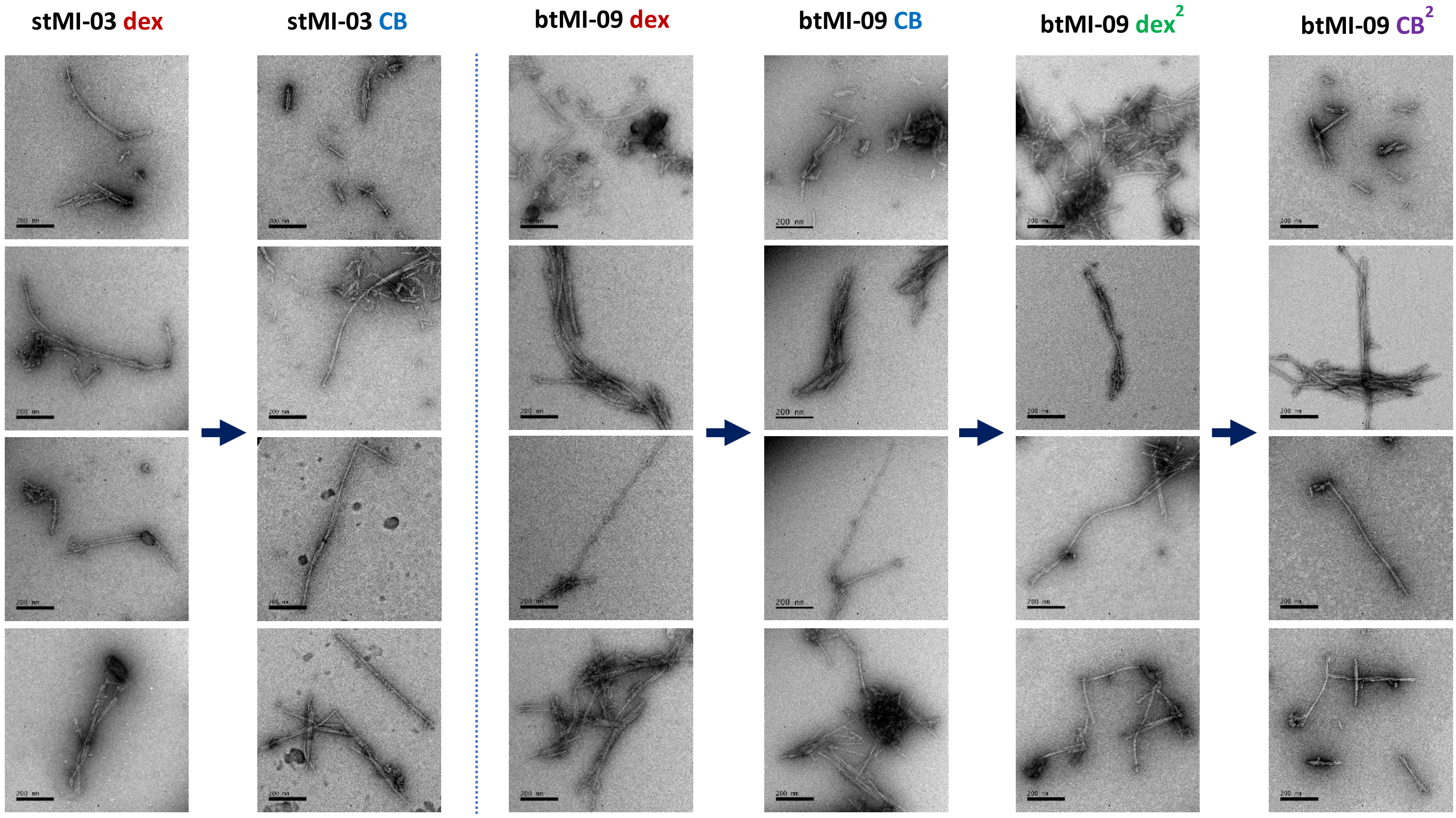

S12 Fig. Negative staining electron microscopy micrographs of PMSA products stMI-03 and btMI-09 propagated in cofactor-free substrate, and btMI-09 back-passaged to dextran sulfate-complemented substrate and again to a cofactor-free environment. The images display four representative micrographs for each of the following PMSA products: stMI-03 dex (original preparation), stMI-03 CB, adapted to a cofactor-free substrate, btMI-09 dex (original preparation), btMI-09 CB (adapted to a cofactor-free substrate), btMI-09 dex^2 (resulting from the back-passage of btMI-09 CB to a dextran-complemented substrate), and btMI-09 CB^2 (the product of readapting btMI-09 dex^2 to a substrate without cofactor). Samples were partially purified through ultracentrifugation in a density gradient, stained with uranyl acetate, and imaged with a transmission electron microscope JEM-1230 (JEOL) at 100 kV, equipped with a CCD Orius SC1000 (GATAN) camera. All PMSA products show fibrillar structures reminiscent of brain-derived prion rods, with no notable differences from the original dextran-complemented products that could explain the observed changes in biochemical and biological features. In all cases, the most remarkable structural features previously observed are conserved upon propagation in different substrates, including rods with two parallel axial densities, presence of straight and curved fibers, unidentified electrodense material, and high propensity for lateral clustering. Each image in the group of four contains a link that opens a higher resolution version in a web browser when clicked.
