# Supplementary material for: Cofactors facilitate bona fide prion misfolding in vitro but are not necessary for the infectivity of recombinant murine prions
Source: PLoS Pathog. 2025 Jan 22;21(1):e1012890. doi: 10.1371/journal.ppat.1012890 (PMC11774496; doi:10.1371/journal.ppat.1012890)
Supplement: S13 Fig — Brain homogenates from C57BL/6 mice inoculated in second passage with the recombinant PMSA products after successful infection of either TgMoL108I or wild type mice in first passage were analyzed by proteinase K (PK) digestion, electrophoresis, and Western blot (Sha31, 1:4,000). Results revealed the presence of indistinguishable classical three-banded PrPSc patterns, characterized by the prominence of the diglycosylated band contrasting with control 22L and RML prions transmitted in the same models. The gel shows one representative sample from each group inoculated with different recombinant products, alongside brain-derived RML and 22L strains. PK: Proteinase K; NBH: Undigested normal brain homogenate; MW: Molecular weight marker. (PDF) [file ppat.1012890.s014.pdf]

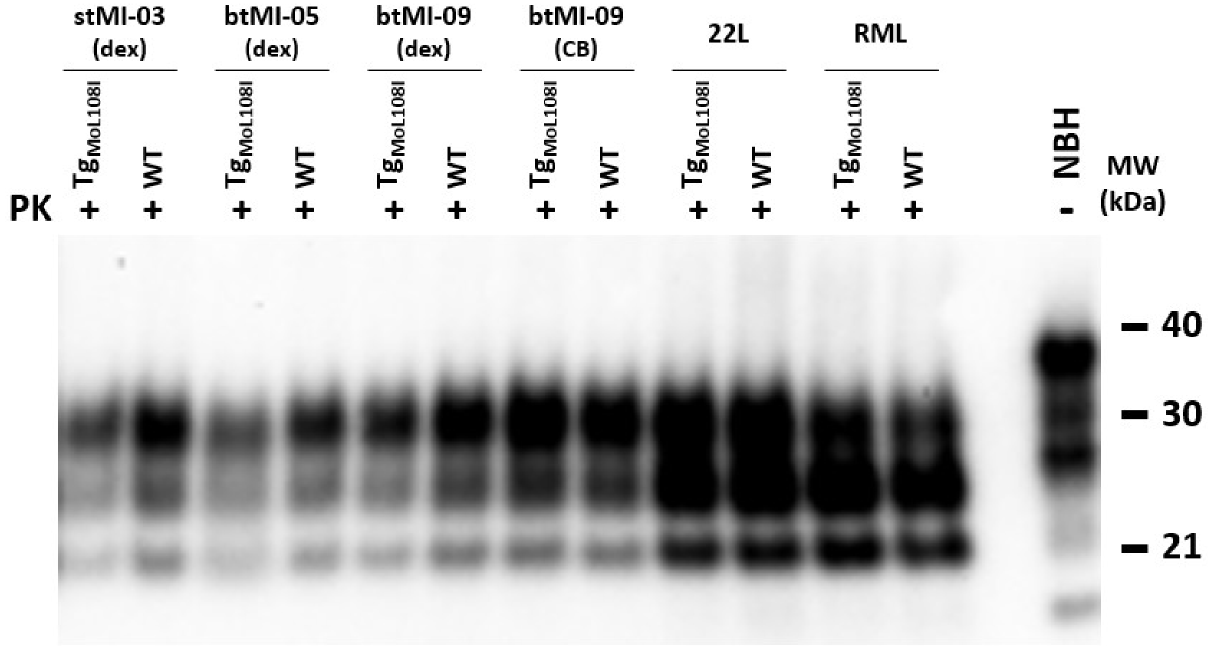

S13 Fig. Biochemical analysis of secondary transmissions of spontaneously misfolded PMSA products to wild-type mice. Brain homogenates from C57BL/6 mice inoculated in second passage with the recombinant PMSA products after successful infection of either TgMoL108I or wild type mice in first passage were analyzed by proteinase K (PK) digestion, electrophoresis, and Western blot (Sha31, 1:4,000). Results revealed the presence of indistinguishable classical three-banded PrP^{Sc} patterns, characterized by the prominence of the diglycosilated band contrasting with control 22L and RML prions transmitted in the same models. The gel shows one representative sample from each group inoculated with different recombinant products, alongside brain-derived RML and 22L strains. PK: Proteinase K; NBH: Undigested normal brain homogenate; MW: Molecular weight marker.
