# Supplementary material for: Cofactors facilitate bona fide prion misfolding in vitro but are not necessary for the infectivity of recombinant murine prions
Source: PLoS Pathog. 2025 Jan 22;21(1):e1012890. doi: 10.1371/journal.ppat.1012890 (PMC11774496; doi:10.1371/journal.ppat.1012890)
Supplement: S14 Fig — The images display sixteen representative micrographs for each of the PMSA products MoL108I-CB-01 and MoL108I-CB-02 after partial purification through ultracentrifugation in a density gradient. Partially purified samples were stained with uranyl acetate and imaged with a transmission electron microscope JEM-1230 (JEOL) at 100 kV, equipped with a CCD Orius SC1000 (GATAN) camera. The two PMSA products show fibrillar structures reminiscent of brain-derived prion rods, with indistinguishable ultrastructural features. As in all previous preparations characterized by TEM, clusters of unidentified electrodense material (Em) were observed near the rods. Whether these are contaminants from the purification process or biologically significant for fiber formation is yet to be determined. Despite their high similarity to previous dextran-complemented preparations, with many fibers showing two parallel axial densities of approximately 12 nm resembling rail tracks (parallel yellow lines), lateral clustering of fibers and arrangement in the form of bundles is apparently lower (pointed by yellow lines), as is the presence of curved fibers (C, in yellow). In contrast, the presence of torsions (indicated by yellow arrows) or twisted fibers was higher than in previous preparations. Each image in the group of sixteen contains a link that opens a higher resolution version in a web browser when clicked. (PDF) [file ppat.1012890.s015.pdf]

# MoL108I-CB-01

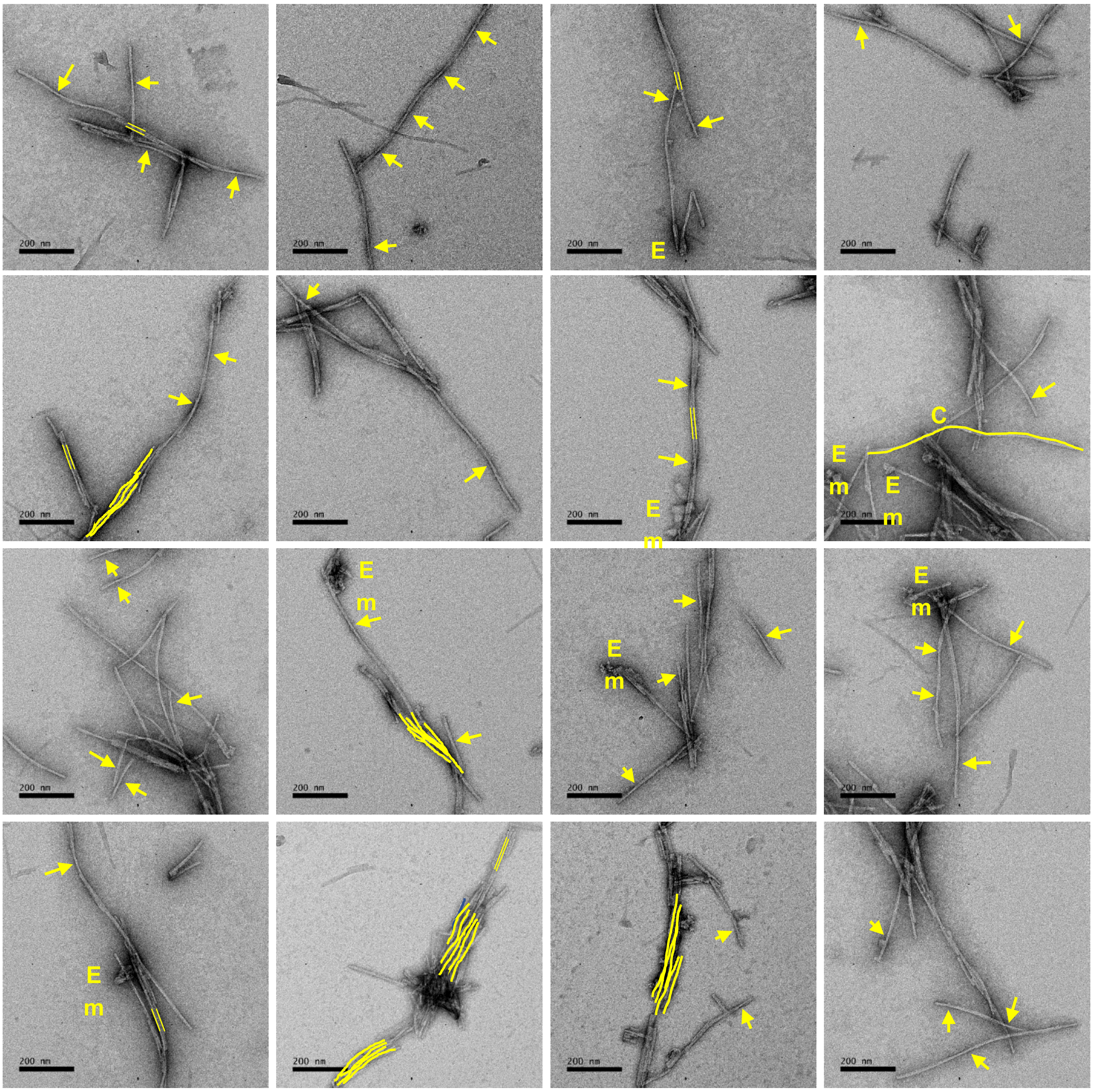

## MoL108I-CB-02

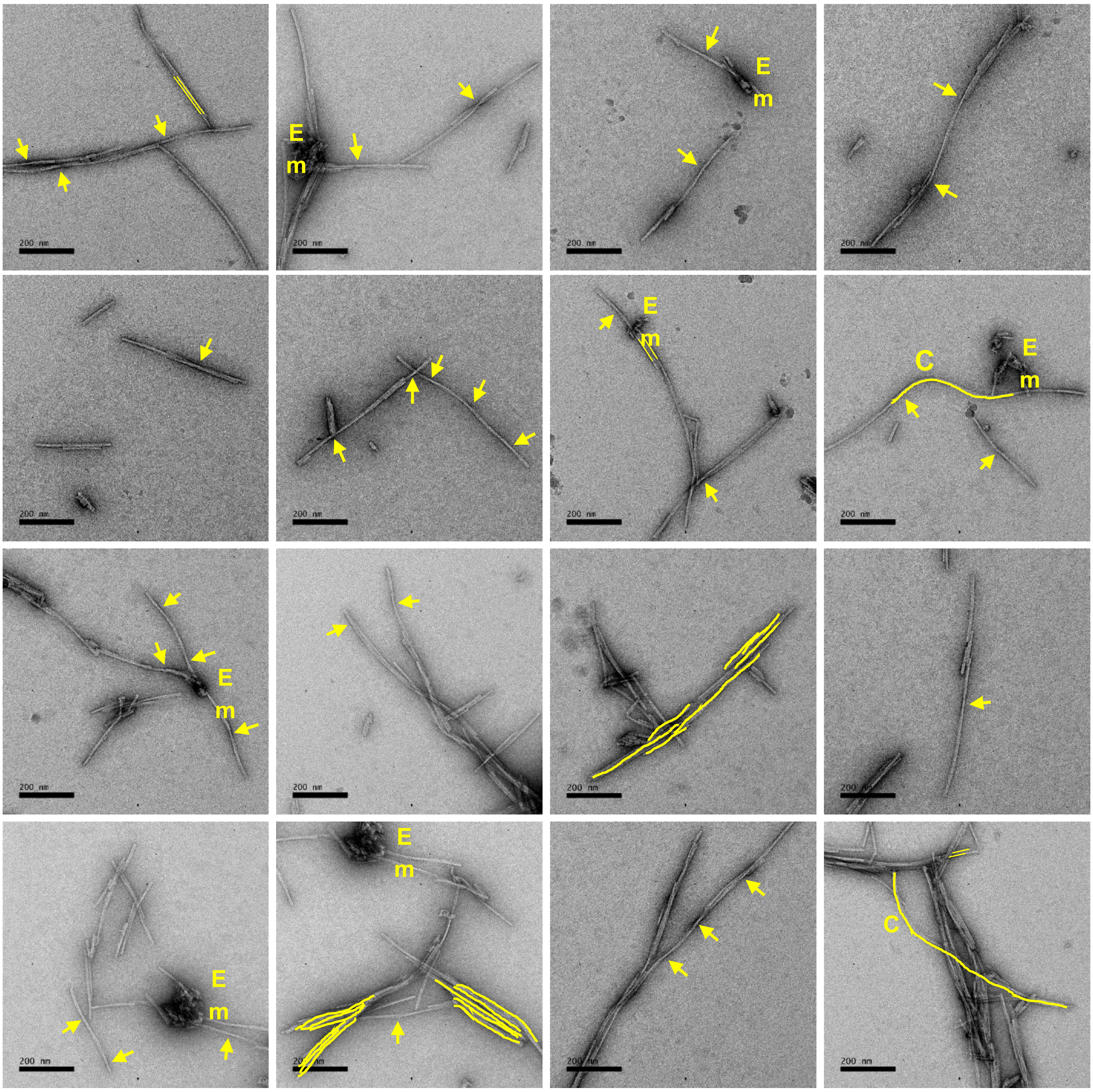


**S14 Fig. Negative staining electron microscopy micrographs of two PMSA products generated spontaneously in the absence of cofactor.** The images display sixteen representative micrographs for each of the PMSA products MoL108I-CB-01 and MoL108I-CB-02 after partial purification through ultracentrifugation in a density gradient. Partially purified samples were stained with uranyl acetate and imaged with a transmission electron microscope JEM-1230 (JEOL) at 100 kV, equipped with a CCD Orius SC1000 (GATAN) camera. The two PMSA products show fibrillar structures reminiscent of brain-derived prion rods, with indistinguishable ultrastructural features. As in all previous preparations characterized by TEM, clusters of unidentified electrodense material (Em) were observed near the rods. Whether these are contaminants from the purification process or biologically significant for fiber formation is yet to be determined. Despite their high similarity to previous dextran-complemented preparations, with many fibers showing two parallel axial densities of approximately 12 nm resembling rail tracks (parallel yellow lines), lateral clustering of fibers and arrangement in the form of bundles is apparently lower (pointed by yellow lines), as is the presence of curved fibers (C, in yellow). In contrast, the

presence of torsions (indicated by yellow arrows) or twisted fibers was higher than in previous preparations. Each image in the group of sixteen contains a link that opens a higher resolution version in a web browser when clicked.
