# Supplementary material for: Cofactors facilitate bona fide prion misfolding in vitro but are not necessary for the infectivity of recombinant murine prions
Source: PLoS Pathog. 2025 Jan 22;21(1):e1012890. doi: 10.1371/journal.ppat.1012890 (PMC11774496; doi:10.1371/journal.ppat.1012890)
Supplement: Attachment — Submitted filename: Response to Reviewers.docx [file ppat.1012890.s016.docx]

Dear Editors,

Thank you for selecting our manuscript for revision. We have addressed all the points raised by the reviewers, as detailed in the present document. We expect that our responses to all the issues raised by the reviewers are satisfactory and allow the publication of this improved version of the original article. Changes in the main text and figure legends have been marked in blue in the re-submitted version of the manuscript and Supplementary material file.

**Reviewers' Comments:**

 Reviewer's Responses to Questions

**Part I - Summary**
Please use this section to discuss strengths/weaknesses of study, novelty/significance, general execution and scholarship.

Reviewer #1: The manuscript by Pérez-Castro et al. presents extensive data on the spontaneous generation of infectious prions from recombinant PrP via Protein Misfolding Shaking Amplification (PMSA) in different environments, including dextran sulfate as a cofactor. Their findings challenge previous assertions by demonstrating that cofactors are not essential for acquisition of infectivity or strain characteristics.

The study is comprehensive and integrates data effectively from multiple experimental models. However, given the substantial amount of data and to maintain clarity and ensure accessibility, the presentation could benefit from simplified / more descriptive legends to aid readability. Some sections, particularly those comparing the neuropathological properties of the recombinant prion strains, are particularly dense and could be streamlined.
To strengthen the interpretation of the data, further clarification on controls and statistical methods is necessary.

We would like to thank the reviewer for the dedication to reviewing this unusually long work. We really appreciate all the comments intended to improve the present manuscript and its readability. We will carefully consider all the comments and try to clarify all the issues raised. Apart from addressing the specific comments below, we have made an effort to reduce some sections of the manuscript with special attention to figure legends. Regarding the strengthening the data interpretation, we have included statistical analysis to unveil whether differences in incubation periods are statistically significant and could help determine strain differences, as explained in detail below.

Reviewer #2: The manuscript by Pérez-Castro et al. describes novel synthetic prions generated by mutated mouse prion protein amplified in vitro and then passaged in vivo in various hosts. The conclusions drawn by this work is that generation of synthetic prions occurs without the need of cofactors.

We thank the reviewer for the accurate summary of our work and appreciate the efforts to improve the manuscript.

Reviewer #3: Pérez-Castro and colleagues present an analysis of factors involved (or not) in allowing spontaneous in vitro formation of infectious prions. In an impressive array of in vitro prion propagation reactions and in vivo bioassays, they provide strong evidence that infectious prions can be formed spontaneously under very simple conditions in the absence of polyanionic cofactors. The work is quite thought-provoking and addresses fundamental issues of prion biology and biochemistry. It should be of real significance to the prion field. The presentation and interpretation of the results is thorough, nuanced and clear, for the most part (see below). However, I have some suggestions for improving the manuscript for the authors’ consideration.

We would like to sincerely thank the reviewer for the thorough evaluation and constructive comments We will try to address all concerns raised to the best of our ability.

**Part II – Major Issues: Key Experiments Required for Acceptance**
Please use this section to detail the key new experiments or modifications of existing experiments that should be absolutely required to validate study conclusions.

Generally, there should be no more than 3 such required experiments or major modifications for a "Major Revision" recommendation. If more than 3 experiments are necessary to validate the study conclusions, then you are encouraged to recommend "Reject".

Reviewer #1: 1. Bioassays in different animal models.
- The study includes limited serial passages (one or two at most) within the same host model, i.e. without full terminal adaptation or stabilization of optimized conformers. This likely contributes to variability in neuropathological findings (e.g., lines 640-660 and elsewhere) and western blot analyses. Acknowledging this in the discussion would add context to the observed inconsistencies in strain stability.

Indeed, two serial passages were performed at most in order to characterize the recombinant prions generated in PMSA, which as stated by the reviewer, may affect strain adaptation and stability. As suggested by the reviewer, we have now acknowledged in the Discussion section (lines 1189-1202) that this likely contributes to the heterogeneity observed in neuropathological findings and biochemical analyses.

*However, in our case, the fact that some preparations could show similarity in some models while differences in others, together with the variability found in a single group of animals, especially for stMI-01 and stMI-03, suggest that the phenotypic differences are independent of host-exclusive factors and more likely related to conformer mixtures and their relative stability. Nonetheless, since no more than two serial transmission experiments were performed in the different animal models for each PMSA product, we cannot exclude the possibility that they are not fully adapted or stabilized to the brain environment, requiring additional passages for that purpose. This fact could also contribute to the heterogeneity of the neuropathological lesions found within some groups and could also have an impact on the final lesion profiles characteristic of each preparation, that could be slightly different from those of the fully adapted strains. Thus, considering cautiously the results from the second passages and attending to the distinct rodent prion strain classes based on their stability upon transmission in vivo [76], btMI-05 and especially btMI-09 are reminiscent of a Class II strain, highly stable in the same host, altered when inoculated in a different host, but able to stabilize rapidly in the new environment.*

- The tgMoL108I and tgVole 1x mouse models (I109) are not adequately referenced, leaving some uncertainty about the models’ documented behavior and the occurrence of spontaneous prion disease. Providing background on these models would increase confidence in the results.
For instance, the initial bioassay testing the infectivity of the stMI-01, stMI-03, btMI-05, and btMI-09 preparations in tgMoL108I mice mentions that these mice spontaneously accumulate prions with age, which are non-infectious to wild-type mice. This statement lacks a supporting reference, and inclusion of a control experiment using mock-inoculated tgMoL108I mouse brain in wild-type mice would clarify the claim and confirm any impact on the findings from the second passage in wild-type mice.

The reviewer is right on pointing out that there is no reference to previous publications for the TgMoL108I mouse model. Unfortunately, we haven’t published any study performed with this model before in any peer-reviewed journal. We agree that it may not be sufficient with the statement indicating the lack of transmissibility of spontaneous TgMOL108I disease to WT mice, so we have included the requested data.

Table 2 has been modified to include a new row with the second passage of non-inoculated TgMoL108I terminal brains in wild-type mice. Additionally, a new supplementary figure (new Supplementary figure 5) has been included showing the histopathological evaluation of these wild-type mice inoculated sequentially with the brain homogenate of a spontaneously sick TgMoL108I mice. These additions provide experimental support for our statement about the non-transmissibility of spontaneous TgMoL108I disease to wild-type mice.

- Comparisons between direct transmission to wild-type mice and two-passage transmission via tgMoL108I mice suggest distinct strain properties. However, these different transmission paths introduce potential confounders. Sequential barriers and strain competition may influence conformer stability and adaptation, and acknowledging this complexity in the discussion would provide a more nuanced interpretation of the data.

We agree with the reviewer that for the strain typing of the recombinant prion preparations, serial passages across different models could introduce confounding factors. Although this possibility was briefly mentioned in the Results of the transmission experiments, we consider important that this is completely clear and, as suggested by the reviewer, we have now expanded this discussion by adding a new paragraph in lines 1182-1188:

*In any case, despite the apparent differences underscored by the inoculation in distinct models, it is important to take into account that TgMoL108I to wild type mouse, wild-type to wild-type, and TgVole to TgVole transmissions have been considered together to highlight differential strain features. Both sequential transmission barriers and dissimilar strain competition could have influenced the results, making strain typing of recombinant preparations a complex issue that precludes robust conclusion on the final number of distinct strains.*

- The authors note a significant reduction in incubation periods between the first and second passages when they transmitted stMI-01, stMI-03, btMI-05 and btMI-09 to wild-type mice, which they consider "extraordinary." However, reductions of this magnitude are common in prion studies. Additionally, to confirm stability and adaptation, a third passage may be necessary to observe full strain stabilization.

As pointed out by the reviewer reduction in incubation periods is a usual finding in prion transmission studies and thus, our observations are not extraordinary in that sense. We got carried away by the short incubation periods observed in wild type animals with respect to other synthetic prions generated in the past. We have modified the phrase to eliminate the term ‘extraordinary’.

Regarding stability and adaptation, we also agree that we cannot ensure it with the present data and thus, we have been cautious on addressing this point. Unfortunately, due to timing and funding constrains, we were unable to perform a third passage to ensure full strain stabilization. Nonetheless, we believe that the results shown are sufficient to conclude the existence of distinctive features in the PMSA preparations, and more importantly the analysis of second passages only, does not invalidate the general conclusions of the article, that infectious prions can be obtained in absence of polyanionic cofactors and that their presence has some influence on the pathobiological features of the recombinant prions generated by PMSA. We have added a new sentence acknowledging this limitation in the Results section (lines 724-728):

*This secondary transmission (bottom third of Table 2) resulted in a notable reduction of incubation periods, likely due to overcoming transmission barriers posed by the amino acid sequence mismatch and differences between recombinant and brain-derived PrP, although without a third passage it is not possible to confirm full adaptation and stability of the recombinant prion preparations.*

2. Biochemical analyses

- In comparing the PK resistance of PMSA-generated prions (stMI-01, stMI-03, btMI-05, and btMI-09), the data showing stMI-03’s increased PK resistance is not convincing, as the band at 2 mg/mL is barely visible, and the 1 mg/ml result is unclear due to apparent technical issue. Repeating these analyses with quantitative or statistical studies would strengthen this claim.

The reviewer is right at pointing out that the quality of the gel showed for the PK resistance assay is not optimal and thus, does not allow to conclude differences in PK resistance. As indicated, we have repeated the PK resistance analysis of the four PMSA preparations and apart from showing a new Coomassie gel improved with respect to the previous one, we have included three independent digestions of the same samples visualized by Western Blot. The signal intensity was analysed through densitometry and results plotted in order to quantitatively assess PK resistance. See new supplementary figure 2A, that depicts the new gels and the corresponding plot.

- Directly comparing on the same western blots the PrPres signatures after transmission with and without cofactor, including after PNGase treatment, would provide clearer insights into the structural stability of PMSA-generated prions under different conditions.
For example, Figure 5 displays PrPres profiles after direct transmission to wild-type mice, which show mostly a migration profile higher than with the RML or 22L strains. In tgI108M, the opposite is found. The PrPres signatures after one passage in tgI108M and another in wild-type mice are not shown, why?

We agree with the reviewer that showing the PrP^res^ signatures of the different preparations after transmission into the different models could be informative. Nonetheless, as long as different sizes of the monoglycosilated PrP^res^ fragment are already well discriminated in the current gel, we find the PNGase treatment unnecessary. Adding this analysis would further extend an already comprehensive manuscript.

The PrP^res^ signatures that are not shown throughout the manuscript are those from the secondary transmissions, since we considered at first that providing the detailed lesion profiles would be more valuable than showing again PrP^res^ signatures. Nonetheless, as pointed out by the reviewer, comparison of PrP^res^ signatures after transmission from TgMoL108I to WT and from WT to WT in the same gel, would provide valuable insight into the structural stability of PMSA generated prions across cross-species transmission. In addition, comparison of PrP^res^ signatures after adaptation of originally dextran complemented prions to cofactor-free environment may benefit from direct comparison in the same gel. Thus, addressing the recommendation from the reviewer, we have designed a new supplementary figure showing PrP^res^ signatures after secondary transmission to wild-type mice (both TgMoL108I to WT and WT to WT) for all three preparations that were infectious in this model (stMI-03 dex, btMI-05 dex and btMI-09 dex and for the brain-derived RML and 22L prion strains). The same passages performed with btMI-09 CB are also included to compare with the dextran-complemented strain, to check stability under different conditions (See new supplementary figure 13). A reference to this new supplementary figure has been also included in lines 991-999.

*Additionally, the electrophoretic mobility patterns of these preparations after secondary transmission to wild-type mice (both, secondary transmissions from TgMoL108I to wild type, and those performed directly in wild-type mice) were compared with all the PMSA products that resulted infectious in C57BL/6 mice, revealing no differences among recombinant preparations transmitted to wild-type mice and thus supporting that btMI-09 dex and btMI-09 CB could be identical (Supplementary figure 13). Moreover, this figure shows the convergence of electrophoretic mobility profiles after secondary transmission to wild type mice, despite in some cases differences were detected in first passages to either TgMoL108I (figure 3) or C57BL/6 (figure 5).*

- The role of various proteolytic fragments in PMSA-generated prions is not discussed. Given that the absence of dextran sulfate in PMCA experiments affects the presence of the 16 kDa fragment, discussing its potential contributions to infectivity or strain properties would add depth to the findings.

We agree that the changes in proteolytic fragments after adaptation to cofactor-free environment, particularly the 16 kDa band, warrant further discussion. While we initially focused on this band due to its association with *bona fide* prion preparation in our previous work with bank vole recombinant prions, we were cautious about making extensive interpretations about its structural role. Nonetheless, in agreement with the reviewer’s comment, we have included a new paragraph in the discussion to highlight the possible relevance of the proteolytic patterns in relation to infectivity and strain properties (lines 1232-1253):

*The changes in electrophoretic mobility patterns of both PMSA products after adaptation to a cofactor-free environment raised questions about the relationship between specific fragments and the infectivity or strain properties of different recombinant prions. Initially, the presence of an approximately 16 kDa band, corresponding to the amyloid core after N-terminal digestion in brain-derived prions [78], was considered essential for in vivo infectivity. This band was used as a selection criterion to confirm rec-PrP misfolding in dextran sulfate-supplemented PMSA reactions [45], as our previous work with bank vole rec-PrP strongly suggested it indicated bona fide prion misfolding [46]. Nonetheless, the infectivity of btMI-09 adapted to CB despite lacking this band suggests that structurally distinct misfolded rec-PrP conformers can act as bona fide prions in cofactor-free conditions. This aligns with the diverse PIRIBS structures found in scrapie-derived rodent prions [4, 6] and those causing Gerstmann-Sträussler-Scheinker syndrome with F198S mutation [79]. Albeit different post-PK electrophoretic profiles typically indicate distinct structural arrangements based on protease accessibility, the similar behavior in TgMoL108I of btMI-09 propagated in the presence and in the absence of dextran suggests other factors, including cofactor binding, may influence prion electrophoretic profiles. This was confirmed when dextran sulfate prevented complete digestion of the 16 kDa fragment in btMI-09 CB and CB2 preparations, consistent with recent structural studies showing electrodense particles (potentially polyanionic cofactors) strongly associated with prion fibrils [4-6, 8, 9]. Alternatively, the adaptation to different environments might have induced subtle conformational changes that significantly affected electrophoretic mobility but minimally impacted pathobiological or strain features, suggesting that distinct conformers could show highly resemblant pathobiological characteristics despite apparent structural differences.*

Reviewer #2: The work is well presented and executed. The manuscript itself is well-written and orderly organized.
While the methods are described in details and the experiments presented with a clear fashion, I only have a more fundamental scientific issue with this work.
Despite the wealth of citations employed in support of the presentation, discussion and conclusions of the work, fundamental previous work is overlooked. I hope that this problem is simple a forgetfulness issue by the authors but nevertheless essential for the readers that may not be accustomed with previous seminal work.
There are several papers that have shown that to obtain synthetic prions one does not need cofactors and these works are not cited in this manuscript.
The following papers several years ago have shown that it is possible to generate wild-type mouse synthetic prions without the use of sequence exchange of any amino acid. It has been described that there is no need to use bank vole sequence to propagate in a more permissive way any synthetic prion.
The papers are:
Synthetic mammalian prions.
Legname G, Baskakov IV, Nguyen HO, Riesner D, Cohen FE, DeArmond SJ, Prusiner SB. Science. 2004 Jul 30;305(5684):673-6. doi: 10.1126/science.1100195.
Strain-specified characteristics of mouse synthetic prions.
Legname G, Nguyen HO, Baskakov IV, Cohen FE, Dearmond SJ, Prusiner SB. Proc Natl Acad Sci U S A. 2005 Feb 8;102(6):2168-73. doi: 10.1073/pnas.0409079102. Epub 2005 Jan 25.
Continuum of prion protein structures enciphers a multitude of prion isolate-specified phenotypes.
Legname G, Nguyen HO, Peretz D, Cohen FE, DeArmond SJ, Prusiner SB. Proc Natl Acad Sci U S A. 2006 Dec 12;103(50):19105-10. doi: 10.1073/pnas.0608970103. Epub 2006 Dec 1.
Design and construction of diverse mammalian prion strains.
Colby DW, Giles K, Legname G, Wille H, Baskakov IV, DeArmond SJ, Prusiner SB. Proc Natl Acad Sci U S A. 2009 Dec 1;106(48):20417-22. doi: 10.1073/pnas.0910350106. Epub 2009 Nov 13.
Protease-sensitive synthetic prions.
Colby DW, Wain R, Baskakov IV, Legname G, Palmer CG, Nguyen HO, Lemus A, Cohen FE, DeArmond SJ, Prusiner SB. PLoS Pathog. 2010 Jan 22;6(1):e1000736. doi: 10.1371/journal.ppat.1000736.
Synthetic prions with novel strain-specified properties.
Moda F, Le TN, Aulić S, Bistaffa E, Campagnani I, Virgilio T, Indaco A, Palamara L, Andréoletti O, Tagliavini F, Legname G. PLoS Pathog. 2015 Dec 31;11(12):e1005354. doi: 10.1371/journal.ppat.1005354. eCollection 2015

We would like to apologize for not including the highly relevant works mentioned above. Indeed, they set the basis for spontaneous synthetic prion obtaining and we agree that they should be included in the manuscript, acknowledging due credit. We initially overlooked them because while they did not require polyanionic cofactors for their formation, in all cases denaturing or reducing agents were used, differing from our system. However, we agree that they should have been included and we have done so, adding specific comments in Introduction and Discussion sections:

Lines 116-128:

*The first of such systems led in fact to the spontaneous generation of bona fide synthetic prions [20] and demonstrated that different conformers could be generated in vitro, showing differential strain features that caused diverse disease phenotypes [21-25], all prepared in the presence of distinct concentrations of denaturing agents such as urea or guanidine hydrochloride.*

*Grounded on these initial cell-free prion propagation systems or amyloid seeding assays [26, 27], further developments led to one of the most widely used tools in this regard, the Protein Misfolding Cyclic Amplification (PMCA), developed over twenty years ago [28]. This system allowed, among other things, the generation of spontaneously misfolded PrP from brain homogenates of wild-type animals used as substrate without the need of using denaturing conditions during the reaction. These prions generated spontaneously in vitro in native conditions, while infectious in vivo, showed an unusually long incubation period and displayed different strain properties compared to previously characterized prion strains [29, 30].*

Lines 153-157:

*This latter hypothesis is also supported by the variable outcome in terms of infectivity mentioned previously for synthetic prions generated in the presence of RNA and POPG, which may allow some degree of strain variability, and the generation of recombinant infectious prions in cofactor-free environment, although this approach required seeding with previously formed prions or the use of denaturing agents [25, 43, 44].*

In addition, what claimed to be a novel technique for generation of in vitro generated synthetic prions, PMSA, is instead a variation of techniques already in use for quite some time in several laboratories, namely amyloid seeding assay (ASA) and real time – quacking induced conversion (RT-QuIC).
The following papers describe these techniques for the first time several years ago:
Prion detection by an amyloid seeding assay.
Colby DW, Zhang Q, Wang S, Groth D, Legname G, Riesner D, Prusiner SB. Proc Natl Acad Sci U S A. 2007 Dec 26;104(52):20914-9. doi: 10.1073/pnas.0710152105. Epub 2007 Dec 20.
Ultrasensitive detection of scrapie prion protein using seeded conversion of recombinant prion protein.
Atarashi R, Moore RA, Sim VL, Hughson AG, Dorward DW, Onwubiko HA, Priola SA, Caughey B. Nat Methods. 2007 Aug;4(8):645-50. doi: 10.1038/nmeth1066. Epub 2007 Jul 22.
I would like to suggest that the authors cite these works accordingly prior to claim that they have found anything novel.
Again, the work is of interest and may deserve to be published but maybe a little biased toward something that is not completely novel.

As pointed out by the reviewer, PMSA is a similar technique to other amyloid seeding assays based on a PrP substrate and an external energy input to induce aggregate formation or propagation, and we agree that as such, it should be noted that it is another ASA. Thus, in agreement with the reviewer’s comment, we have tried to clarify this point, moderating our claims of novelty and acknowledging previous technical developments:

Lines 121-123:

*Grounded on these initial cell-free prion propagation systems or amyloid seeding assays [26, 27], further developments led to one of the most widely used tools in this regard, the Protein Misfolding Cyclic Amplification (PMCA), developed over twenty years ago [28].*

Lines 161-163:

*In this work, we explore the role of a polyanionic cofactor in spontaneous prion generation using Protein Misfolding Shaking Amplification (PMSA). PMSA is a recently developed variant derived from the previous amyloid seeding assays based on the use of recombinant PrP [26, 27] that overcomes some variability issues of PMCA, allowing consistent and swift generation of hundreds of infectious recombinant prions using recombinant PrP and dextran sulfate as a polyanionic cofactor [45, 46].*

Lines 1092-1095:

*Our previous research on in vitro prion misfolding and propagation, grounded in work demonstrating that spontaneous rec-PrP misfolding could be achieved in vitro with minimal components [20, 31], led us to develop the Protein Misfolding Shaking Amplification (PMSA) method, a variant of previous amyloid seeding assays [26, 27].*

Reviewer #3:

1) Lines 491-2: “…However, stMI-03 showed higher resistance up to 2000 μg/ml…” >>This is not apparent in the figure, especially comparing the 1000 ug/ml digestions. In fact, stMI-03 seems to be mostly digested at 1000 ug/ml then, paradoxically, is less digested at 2000. Also, the overall protein load seems to be a bit lower for stMI-03 than the others, confounding such comparisons. This experiment bears repeating, and perhaps, reinterpreting.

We agree with the reviewer that the figure used to illustrate the results for the PK resistance assay is deficient and does not adequately support the conclusions withdrawn from it. As recommended, we have repeated the analysis of the four PMSA preparations and apart from showing a new Coomassie gel improved with respect to the previous one, we have included three independent digestions of the same samples visualized by Western Blot. The signal intensity was analysed through densitometry and results plotted in order to quantitatively assess PK resistance. See new supplementary figure 2A, that depicts the new gels and the corresponding plots. Paragraphs in results and discussion sections have been also modified accordingly.

2) Bioassays: Perhaps I missed it, I was unable to find an accounting of the concentrations of PrP in these inocula, and how those amounts might compare to the concentration of PrPSc in the brain RML and 22L homogenates. This would allow consideration of the important issue of the relative infectivities per mass of PrP when considering the relative incubation periods. Even more informative in this regard would be bioassay data from dilutions of the inocula if available (e.g. if the inocula are diluted 10-fold or more, do the titers become sub-infectious? This would make them much less potent than the brain inocula. In any case, the issue of relative amounts of PrP in the PMSA vs brain inocula should be explained and discussed.

Unfortunately, we did not perform inoculation of serially diluted recombinant preparations to calculate their specific infectivity, assuming a similar behaviour to previous PMSA preparations, that in the case of bank vole recombinant PrP gave rise to a titer of 4.16·10^5^ LD50/ug of misfolded PrP, about 80-fold lower than the CWD-vole strain when inoculated in the same model (see Eraña H et al. Understanding the key features of the spontaneous formation of bona fide prions through a novel methodology that enables their swift and consistent generation. Acta Neuropathol Commun. 2023 Sep 7;11(1):145. doi: 10.1186/s40478-023-01640-8 for further information). In that case, in order to compare the titer of recombinant prions with that of brain-derived isolates, we had to make few assumptions such as the yield of PrP conversion in PMSA (estimated to be close to 100% given that after 24h of reaction all detectable PrP precipitates after centrifugation) and that a rodent brains contains approximately 20 ug of PrPSc per g of brain (based on empiric determination of the prion amount in 263k infected hamster brains by Onisko BC, Silva CJ, Dynin I, Erickson M, Vensel WH, Hnasko R, Requena JR, Carter JM. Sensitive, preclinical detection of prions in brain by nanospray liquid chromatography/tandem mass spectrometry. Rapid Commun Mass Spectrom. 2007;21(24):4023-6. doi: 10.1002/rcm.3310. PMID: 18000838.).

However, after delving deeper into different PMSA reactions with distinct species, we have realized that even though all the rec-PrP originally contained in the substrate precipitates after PMSA and centrifugation, not all of it is protease-resistant and that we cannot rule out the possibility of PK-sensitive species also contributing to the infectivity. Thus, although we know that after PMSA all the rec-PrP precipitates, we don’t know how much of this material corresponds to *bona fide* recombinant prions. In order to circumvent this unresolved issue, we have decided to perform prion amount estimation considering a wide range of conversion yields after PMSA, from 20 to 100 %. So, based on the assumptions that we obtain variable conversion yields in PMSA and that the amount of prions in a terminally sick animal is about 20 ug/g of tissue, we can estimate the amounts of rec-PrP^Sc^ or brain-derived PrP^Sc^ (for RML and 22L) inoculated in animals that would allow direct comparison of the infectivity of the PMSA products and the brain-derived inocula. We have included this general estimation, valid for all the recombinant preparations since the rec-PrP in the substrate does not vary (despite the final yield seems usually slightly lower for preparations not complemented with dextran sulfate), in the first section of results in which inoculation of recombinant products is described (lines 601-611):

*To compare relative infectivity between recombinant preparations and brain-derived prions, misfolded PrP amounts were estimated following methods previously used for bank vole recombinant prions [46]. Assuming variable conversion yield in PMSA ranging from 20 to 100 % and taking into account that the PMSA substrates were prepared with approximately 0.1 mg/ml of rec-PrP, each PMSA preparation would contain 0.02 to 0.1 mg/ml of misfolded rec-PrP, which after dilution in PBS and considering the volume injected in the animals would suppose around 0.04 to 0.2 µg of misfolded rec-PrP. To estimate the prion load in the RML and 22L preparations, we assume that the titer in a prion infected animal at terminal stage is similar to that determined for 263k-infected hamsters, that is of around 20 µg/g of brain tissue [46], and thus, we injected approximately 0.004 µg, that is, 10 to 50-fold less than for the recombinant preparations.*

A comment on this has also been included in the Discussion section (lines 1162-1166):

*This potential mixture that would require further serial in vivo transmissions for full stabilization, among other possible reasons, could explain the apparently lower infectivity titer of the recombinant preparations, in which there could have been up to 10 to 50-fold more of misfolded PrP than in the isolates used as control of brain-derived prions, 22L and RML.*

2) Lines 748-755: Regarding stMI-01 CB, btMI-05 CB, and btMI-09: It would be interesting/relevant to see the PK-resistant banding profiles of these strains if dextran sulfate were added back to these preparations just prior to PK digestion. Multiple lines of evidence (including cryo-EM structures -see refs listed below) suggest that DS and other polyanionic cofactors would bind to the aligned stacks of cationic sidechains in the N-lobe of the ordered core of prion fibrils and would likely block PK cleavage sites in that lobe. Thus, if any of these PMSA products maintained the essential features of the original assemblies formed in the presence of DS, then you might see a restoration of the original PK-resistance just by adding DS prior to PK treatment (analogous to what you saw with the Dex-containing PMSA rounds).

Indeed, the issue raised is extremely important given that electrophoretic profile was one of the criteria evaluated in order to discriminate between potentially different strains in PMSA preparations. Sulfate dextran could be responsible for alterations in the profile without substantial modification of the underlying amyloid core structure if able to block PK cleavage sites due to its likely strong association with fibrils, and thus, this aspect needs to be evaluated. For that reason, we have prepared a new supplementary figure showing the PK digestion of the recombinant preparations propagated in CB that loss the 16 kDa fragment, btMI-09 CB and btMI-09 CB2, with or without the addition of dextran sulfate to the digestion reaction. As illustrated by this new Supplementary figure 7 panel C, addition of dextran exerts a protective effect against the digestion of the flexible N-terminal of the recombinant prions, as the 16 kDa fragment is detectable when the digestion is carried out in presence of this compound. Thus, the alteration of the electrophoretic mobility pattern observed upon adaptation to a dextran-free environment can be artefactual in some cases. Few comments have been introduced throughout the text to report this finding and the methods have been included in the corresponding section. These findings support the reviewer’s hypothesis about dextran sulfate binding to cationic side chains and blocking PK cleavage sites.

Lines 812-816:

*In contrast, stMI-03 CB was characterized by a very intense 16 kDa band with a ladder-like pattern and slightly more intense bands at ~10 and 5 kDa. Nonetheless, given the potential effect of the dextran sulfate on the digestion, as shown in the next section, changes in the electrophoretic pattern could not necessarily indicate a change in strain properties.*

Lines 936-945:

*Further adaptation of btMI-09 dex^2^ to a cofactor-devoid environment (btMI-09 CB^2^) resulted in the loss of the 16 kDa fragment, similar to the original btMI-09 dex to btMI-09 CB transition. ~~Nonetheless, potential differences in the electrophoretic pattern were noted, with slightly more intense low molecular weight fragments in btMI-09 CB compared to btMI-09 CB~~^~~2~~^~~.~~ However, since dextran sulfate, if strongly associated to prion fibers, could affect proteinase K accessibility or activity, changes in electrophoretic mobility as indicators of potential strain differences should be evaluated carefully. In fact, the digestion of btMI-09 CB and btMI-09 CB^2^ in the presence of dextran sulfate (figure 7C) revealed that this compound hinders the digestion of the 16 kDa fragment, detectable only when the digestion was done in the presence of this molecule.*

Lines 1243-1253:

*Albeit different post-PK electrophoretic profiles typically indicate distinct structural arrangements based on protease accessibility, the similar behavior in TgMoL108I of btMI-09 propagated in the presence and in the absence of dextran suggests other factors, including cofactor binding, may influence prion electrophoretic profiles. This was confirmed when dextran sulfate prevented complete digestion of the 16 kDa fragment in btMI-09 CB and CB2 preparations, consistent with recent structural studies showing electrodense particles (potentially polyanionic cofactors) strongly associated with prion fibrils [4-6, 8, 9].*

**Part III – Minor Issues: Editorial and Data Presentation Modifications**
Please use this section for editorial suggestions as well as relatively minor modifications of existing data that would enhance clarity.

Reviewer #1:

1. Ultrastructural data.
   While transmission electron microscopy (TEM) data are presented, their relevance to the study’s primary conclusions is not entirely clear. Emphasizing only the key TEM results directly tied to the study’s main findings would streamline and strengthen the narrative.

The TEM experiments intended to uncover potential differences in the ultrastructural arrangement of the different PMSA products. But as pointed out by the reviewer, the section describing the results of these studies might be unnecessarily long, describing also other elements that are not relevant to the strain typing, such as the presence of electrodense material. Following the recommendation from the reviewer, the section referring to TEM studies has been shortened to 2/3 of its original length.

2. Statistical Analyses:
A clearer discussion on consistency and variability in propagation assays would be beneficial. Statistical analyses (e.g., non-parametric tests to evaluate differences in incubation times, vacuolation profiles, or seeding activities) would provide a more robust comparison of the prion strains.

Given that we used several different studies for typing potential strain differences among PMSA products and some differences were inferred from multiple distinct results, we did not perform statistical analysis for specific parameters such as incubation periods or vacuolation profiles. However, the reviewer is right on pointing out that statistical analysis of our results, for instance when comparing incubation periods could be beneficial for the final conclusions on strain comparisons. For that, we analyzed first the incubation period data using a Saphiro-Wilk test to determine distribution. If all groups showed normal distribution a one-way ANOVA was performed followed by a pot-hoc Tukey test, while for data with non-normal distribution, the Mann-Whitney U test was done. Similarly, results from the relative resistance to PK were analyzed statistically and conclusions included in the text. This information has been conveyed in Materials and Methods section (lines 418-430):

***Statistical Analysis***

*Statistical analyses were performed using GraphPad Prism version 9.0 (GraphPad Software, San Diego, CA, USA). Incubation period data are presented as mean ± standard error of the mean (SEM). Differences in relative proteinase K resistance were analyzed using a Saphiro-Wilk test to assess distribution, followed by Friedman test with Wilcoxon signed-rank tests for post hoc comparisons. Differences in survival times between groups were analyzed using Kaplan-Meier survival curves, and Saphiro-Wilk test was employed to determine data distribution. For experiments in which all groups show normal distribution, multiple group comparisons of post-inoculation times were performed using one-way ANOVA followed by Tukey's post-hoc test. In the cases in which data showed non-normal distribution, statistical significance was determined using Mann-Whitney U test. Statistical significance was set at P < 0.05, in all cases. Sample sizes were determined based on previous similar studies to ensure adequate statistical power while adhering to the principles of reduction in animal experimentation.*

Lines 524-529:

*As shown in Supplementary Figure 2A, both total protein staining and the Western blot of three independent replicates demonstrated high resistance in all four preparations. stMI-01, btMI-05, and btMI-09, showed similar resistance patterns with no statistically significant differences among them. However, stMI-03 exhibited significantly lower relative resistance compared to the other preparations (stMI-01 vs. stMI-03: p-value= 0.013; btMI-05 vs. stMI-03, p-value= 0.013; and btMI-09 vs. stMI-03, p-value= 0.028).*

Lines 611-613:

*As expected, all PMSA products induced transmissible spongiform encephalopathy (TSE) in the inoculated animals, albeit with no statistically significant differences as determined by one-way ANOVA (p-value = 0.226) (Table 1).*

Lines 647-650:

*Secondary transmission of TgMoL108I-passaged recombinant prions to wild-type (C57BL/6) animals revealed further differences among the four inocula, showing statistically significant differences between all the preparations except for stMI-01 and btMI-05 (Mann-Whitney U test, p-value = 0.635) (Table 2).*

Lines 715-717:

*Statistical analysis of their incubation periods revealed significant differences between stMI-03 and the rest (one-way ANOVA, p-value = 0.0084), while no significant difference was observed for btMI-05 and btMI-09 (post-hoc Tukey’s test, p-value = 0.916).*

Lines 728-730:

*All three strains showed incubation periods of around 130 days post inoculation, faster than any known mouse-adapted prion strain, with no statistically significant differences according to the Mann-Whitney U test results.*

Lines 751-753:

*As shown in Table 3, all four recombinant prion strains induced disease in all inoculated animals, albeit with notable differences in incubation periods, statistically significant for most comparisons except for btMI-05 vs. btMI-09 (p-value =0.349 according to Mann-Whitney U test).*

Lines 765-768:

*Upon second passage, incubation periods of the originally recombinant prions were remarkably reduced, converging to times comparable with those of RML and 22L prions (Table 3), and with statistically significant differences only found between stMI-01 and btMI-05-inoculated animals (p-value = 0.023, Mann-Whitney U test).*

Lines 834-841:

*As shown in Table 4, after adaptation to a cofactor-devoid environment, stMI-03 CB was unable to cause clinical disease in wild-type mice after more than 600 dpi, being significantly distinct to stMI-03 dex (p-value= 0.363, Mann-Whitney U test). In the case of TgMoL108I, despite showing also statistically significant differences with the original dextran-complemented preparation (p-value= 0.0008, Mann-Whitney U test) and the btMI-09-derived preparations, given that the incubation period exceeded the time at which the animals of this transgenic line start developing a spontaneous and atypical prion disease, it was also deemed non-infectious, since their biochemical analysis revealed absence of classical three-banded pattern PrP^Sc^.*

Lines 882-887:

*The study on animals inoculated with btMI-09 CB confirms its capacity to induce a bona fide prion disease in TgMoL108I, in which no statistically significant differences were found in comparison to btMI-09 dex (p-value= 0.118, Mann-Whitney U test); wild-type mice, that revealed significant differences between the two groups (p-value= 0.011); and TgVole (1x), in which the difference resulted non-significant according to Tukey’s post hoc test (p-value= 0.884); showing differential features in each model (Supplementary Figure 10A).*

Lines 951-954:

*In both cases, a prolongation of the incubation periods with respect to btMI-09 dex and btMI-09 CB was detected, which could also indicate some alteration of strain properties although it was only statistically significant when comparing btMI-09 dex and btMI-09 CB^2^ (p-value= 0.035, Mann-Whitney U test).*

Lines 1028-1038:

*These spontaneously generated misfolded and PK-resistant PMSA products were intracerebrally inoculated in TgMoL108I and wild-type mice to assess their infectivity in vivo. As shown in Table 6, both preparations showed 100 % attack rates and similar incubation periods in the case of TgMoL108I, ~~comparable to the recombinant preparations obtained in the presence of dextran sulfate in the same model~~ being the difference statistically no significant between them (p- value= 0.682, Mann-Whitney U test) (Table 1). In wild-type animals, MoL108I-CB-01 also showed complete attack rates and, in this case, statistically significant differences with stMI-03 CB (p-value= 0.001) and btMI-09 CB (p-value= 0.011) preparations. Upon secondary transmission to the same model, incubation times were significantly reduced, reaching 136 ± 3 dpi and showing no statistically significant differences with respect to btMI-09 dex or btMI-09 CB inoculated in the same model, demonstrating also the transmissibility of these recombinant prion generated in absence of cofactor and an incubation period in line with other brain-derived murine prion strains.*

Reviewer #2: N/A

Reviewer #3:

1) Although I appreciate the thorough and nuanced explanations and interpretations, the manuscript is rather long and might become more digestible to readers if redundancies and other less vital verbiage were distilled down a bit.

As recommended, we have tried to eliminate redundancies and explicative paragraphs that were not strictly necessary to understand the work described in the manuscript. See deleted sections throughout the text. According to our estimations, we have reduced the original manuscript around 15 %.

2) Line 82: Add references to now-solved strain-dependent conformations? See why below.

In agreement with the accurate recommendation from the reviewer, we have added the references describing the solved structures of distinct prion strains.

3) Lines 222-223: Unclear. Is CB referring to a buffer or a substrate in a buffer? The composition of CB is critical in understanding appreciating the simplicity of the authors’ conditions. I would suggest describing it in on first mention in the Results, too. I had to spend a bit of time trying to track it down in the Methods.

Conversion buffer or CB is the buffer used for preparing different PMSA substrates, a solution with 0.15 M of NaCl and 1 % triton-X-100 both diluted in PBS. The rest of the components of PMSA substrates, rec-PrP and for cofactor complemented substrates the dextran sulfate are diluted in this buffer for the preparation of the final substrates. Thus, CB would be a buffer, that together with rec-PrP and, in some cases, other cofactors (such as dextran sulfate) conform the PMSA substrate. We have tried to clarify it in Materials and Methods section, and as suggested, we have included an additional definition in Results section.

Lines 227-231: *“Two different recombinant PMSA substrates were prepared: those complemented with 0.5 % dextran sulfate 6,500-10,000 kDa (Sigma-Aldrich) (also referred as Dx or dex in some figures and text) and those prepared without the addition of any specific cofactor (designated as CB, since they contain just the conversion buffer which include 0.15M of NaCl and 1 % Triton-X-100 and recombinant protein).”*

Lines 798-802: *“To explore the role of the cofactor in infectivity and strain characteristics, we propagated the four distinct recombinant mouse prion strains in a PMSA substrate without dextran sulfate, using only conversion buffer (CB, the same buffer used for the preparation of dextran complemented substrates, containing 0.15M of NaCl and 1 % Triton-X-100 diluted in PBS) and recombinant mouse PrP with isoleucine at position 108.”*

4) Line 457: How large were PK-resistant products generated with the histidine, lysine and methionine-containing constructs?

All variants showed similar patterns, with an approximately 16 kDa band, two intermediate bands of around 9-10 and 7-8 kDa, and variable small molecular weight fragments below the 5 kDa marker. The main differences were in the relative intensities of these most prominent bands, L108H was similar to L108I, showing a very faint lower intermediate band but a clear double band below the 5 kDa marker. L108K and L108M products show more balanced intermediate bands of similar intensities and broader bands below 5 kDa, likely indicating a continuum of small fragments. However, these subtle differences did not affect the size of the main bands (16, 10 and 8 kDa), which remained constant across all PMSA products. We have included a brief comment on this in lines (488-493).

*The rec-PrP^res^ resulting from the proteins bearing histidine, lysine, and methionine showed a highly coincidental electrophoretic mobility pattern with the isoleucine-bearing PMSA products. All displayed a predominant fragment of approximately 16 kDa and two intermediate fragments of around 10 and 8 kDa, with the latter appearing fainter in L108H and L108I compared to the other two proteins. Additional small molecular weight fragments below 5 kDa were observed, showing greater variability among different preparations.*

5) Table 1 and line 587: I found it confusing for the attack rate for the 'no inoculum' group to be reported as 0/18 when they showed clinical disease as well as PrPres (even if the banding profile was unusual). It is important to know what proportion of these animals displayed these clinical signs as reflected in the stated average incubation period.

Initially, we reported the attack rate of non-inoculated TgMoL108I animals as 0 out of 18 to distinguish between spontaneous and inoculation-induced disease. However, we agree this could be misleading. For clarity, we have modified the table to show that 100 % of these overexpressing mice develop spontaneous disease, and added an explanatory note in the table legend.

6) Line 1018: show should be showing?

Typo corrected.

7) Lines 1052-3 (and elsewhere): I would recommend that the authors consider these mechanisms in terms of the known cryo-EM structures of ex vivo prions, e.g. PMID: 34433091, PMID: 35831291, PMID: 36342968, PMID: 35831275, PMID: 36646960, and PMID: 39448454. In these papers one can see that the sidechains of residues analogous to bank vole residues 109 and 112, as well as 139 in the mouse- and hamster-adapted scrapie structures, interact closely to form tightly packed H-phobic cores that are likely to be important in stabilizing the overall fibril structure. Differences in the sidechain geometries of V, I, L and M residues likely influences how well they can pack (or not) to form this core, and how readily a mismatched PrPC molecule can add on to a preexisting template (or at least your data would seem consistent with that hypothesis). Also notable is the fact that residues 109 and 112 are near/within a cluster of basic residues with outwardly oriented sidechains that appear to interact with polyanionic cofactors (such as dextran sulfate) in a way that, as noted above, are likely to mask potential PK-cleavage sites.

We really appreciate the recommendation to discuss our results on the light of the latest structural data. We agree that it enriches and provides an important perspective to the findings of the study. As suggested, we have included a new paragraph in Discussion to include this structural point of view. See lines 1141-1150:

*Further investigations into the mechanisms by which this residue leads to these unique effects on PrP misfolding could be highly relevant to deciphering prion conversion at a molecular level. Along this line, the recently solved high resolution structures of distinct rodent prion strains from hamster and mouse prions [4-7, 9], which differ from bank vole PrP by only a few amino acids, show that residues equivalent to 109, 112, and 139 are part of highly packed hydrophobic stretches critical for prion fiber stability. Therefore, alterations in those residues, already proven to be relevant by previous studies, could strongly influence the formation of these critical hydrophobic cores and the fitting of non-homologous PrP^C^ in said positions. According to our results, isoleucine would be favorable in these positions.*

8) Line 1068: Following from the previous comment, PMID: 36342968 is paper that is also particularly pertinent to the current manuscript because it compares prion strains produced in the same genotype of mouse (where the panel of potential cofactors are the same), unlike the case in ref 40 (as nice as ref 40 is).

Indeed, the indicated reference is highly relevant for the topic under discussion, and we completely agree that it should be cited when referring to structural variations of prion strains. We have included this reference as suggested.

9) Line 1142: “thus, cannot be considered an integral component of prions.” >>maybe this should be softened to say "cannot be considered an integral component of ALL prions'?

As correctly pointed out, our results seem to suggest that the effect of the dextran sulfate on the strain properties is different for the two PMSA preparations tested (03 and 09), being apparently more influential for one than the other. Thus, the need of cofactor in the propagation environment to induce misfolding could be also strain dependent. In agreement with this, we have modified the sentence as suggested.

10) Line 1151: loss should be lost?

Typo corrected.

PLOS authors have the option to publish the peer review history of their article ([what does this mean?](https://track.editorialmanager.com/CL0/https:%2F%2Fjournals.plos.org%2Fplospathogens%2Fs%2Feditorial-and-peer-review-process%23loc-peer-review-history/1/010f0192f663e310-921b0dd7-a065-43f5-9621-38c6838fc7eb-000000/mNNfXvtYs6z0zX6YW0TPFqD9dku4aWUBffGDuLWIYgI=183)). If published, this will include your full peer review and any attached files.


If you choose “no”, your identity will remain anonymous but your review may still be made public.


**Do you want your identity to be public for this peer review?** For information about this choice, including consent withdrawal, please see our [Privacy Policy](https://track.editorialmanager.com/CL0/https:%2F%2Fwww.plos.org%2Fprivacy-policy/1/010f0192f663e310-921b0dd7-a065-43f5-9621-38c6838fc7eb-000000/TSV8lEnQ1t4TIfle8_BGkY2CIU6WJRUC9GlxqeQwQQk=183).

Reviewer #1: No

Reviewer #2: No

Reviewer #3: **Yes:** Byron Caughey

[NOTE: If reviewer comments were submitted as an attachment file, they will be attached to this email and accessible via the submission site. Please log into your account, locate the manuscript record, and check for the action link "View Attachments". If this link does not appear, there are no attachment files.]

**Figure resubmission:**

While revising your submission, please upload your figure files to the Preflight Analysis and Conversion Engine (PACE) digital diagnostic tool, <https://pacev2.apexcovantage.com/>. PACE helps ensure that figures meet PLOS requirements. To use PACE, you must first register as a user. Registration is free. Then, login and navigate to the UPLOAD tab, where you will find detailed instructions on how to use the tool. If you encounter any issues or have any questions when using PACE, please email PLOS at [figures@plos.org](mailto:figures@plos.org). Please note that Supporting Information files do not need this step. If there are other versions of figure files still present in your submission file inventory at resubmission, please replace them with the PACE-processed versions.

**Reproducibility:**

To enhance the reproducibility of your results, we recommend that authors of applicable studies deposit laboratory protocols in protocols.io, where a protocol can be assigned its own identifier (DOI) such that it can be cited independently in the future. Additionally, PLOS ONE offers an option to publish peer-reviewed clinical study protocols. Read more information on sharing protocols at <https://plos.org/protocols?utm_medium=editorial-email&utm_source=authorletters&utm_campaign=protocols>
